# Rumen and hindgut microbiome regulate average daily gain of preweaning Holstein heifer calves in different ways

**DOI:** 10.1186/s40168-024-01844-7

**Published:** 2024-07-19

**Authors:** Sheng-yang Xu, Xiao-ran Feng, Wei Zhao, Yan-liang Bi, Qi-yu Diao, Yan Tu

**Affiliations:** Institute of Feed Research, Chinese Academy of Agricultural Sciences/Sino-US Joint Lab On Nutrition and Metabolism of Ruminant/Key Laboratory of Feed Biotechnology of the Ministry of Agriculture and Rural Affairs, Beijing, 100081 People’s Republic of China

**Keywords:** Gastrointestinal microbiome, Gastrointestinal metabolome, Host metabolome, Holstein heifer calves, Preweaning ADG

## Abstract

**Background:**

The average daily gain (ADG) of preweaning calves significantly influences their adult productivity and reproductive performance. Gastrointestinal microbes are known to exert an impact on host phenotypes, including ADG. The aim of this study was to investigate the mechanisms by which gastrointestinal microbiome regulate ADG in preweaning calves and to further validate them by isolating ADG-associated rumen microbes in vitro.

**Results:**

Sixteen Holstein heifer calves were selected from a cohort with 106 calves and divided into higher ADG (HADG; *n* = 8) and lower ADG (LADG; *n* = 8) groups. On the day of weaning, samples of rumen contents, hindgut contents, and plasma were collected for rumen metagenomics, rumen metabolomics, hindgut metagenomics, hindgut metabolomics, and plasma metabolomics analyses. Subsequently, rumen contents of preweaning Holstein heifer calves from the same dairy farm were collected to isolate ADG-associated rumen microbes. The results showed that the rumen microbes, including *Pyramidobacter* sp. C12-8, *Pyramidobacter* sp. CG50-2, *Pyramidobacter porci*, *unclassified_g_Pyramidobacter*, *Pyramidobacter piscolens*, and *Acidaminococcus fermentans*, were enriched in the rumen of HADG calves (LDA > 2, *P* < 0.05). Enrichment of these microbes in HADG calves’ rumen promoted carbohydrate degradation and volatile fatty acid production, increasing proportion of butyrate in the rumen and ultimately contributing to higher preweaning ADG in calves (*P* < 0.05). The presence of active carbohydrate degradation in the rumen was further suggested by the negative correlation of the rumen microbes *P. piscolens*, *P.* sp. C12-8 and *unclassified_g_Pyramidobacter* with the rumen metabolites D-fructose (*R* <  − 0.50, *P* < 0.05). Widespread positive correlations were observed between rumen microbes (such as *P. piscolens*, *P. porci*, and *A. fermentans*) and beneficial plasma metabolites (such as 1-pyrroline-5-carboxylic acid and 4-fluoro-L-phenylalanine), which were subsequently positively associated with the growth rate of HADG calves (*R* > 0.50, *P* < 0.05). We succeeded in isolating a strain of *A. fermentans* from the rumen contents of preweaning calves and named it *Acidaminococcus fermentans* P41. The in vitro cultivation revealed its capability to produce butyrate. In vitro fermentation experiments demonstrated that the addition of *A. fermentans* P41 significantly increased the proportion of butyrate in the rumen fluid (*P* < 0.05). These results further validated our findings. The relative abundance of *Bifidobacterium pseudolongum* in the hindgut of HADG calves was negatively correlated with hindgut 4-hydroxyglucobrassicin levels, which were positively correlated with plasma 4-hydroxyglucobrassicin levels, and plasma 4-hydroxyglucobrassicin levels were positively correlated with ADG (*P* < 0.05).

**Conclusions:**

This study’s findings unveil that rumen and hindgut microbes play distinctive roles in regulating the preweaning ADG of Holstein heifer calves. Additionally, the successful isolation of *A. fermentans* P41 not only validated our findings but also provided a valuable strain resource for modulating rumen microbes in preweaning calves.

Video Abstract

**Supplementary Information:**

The online version contains supplementary material available at 10.1186/s40168-024-01844-7.

## Background

Dairy cows play a vital role in meeting the increasing demand for high-quality protein worldwide. Given that calves serve as the future asset for dairy herds, the quality of calf rearing holds utmost significance in the dairy industry. Numerous studies have highlighted that enhancing preweaning average daily gain (ADG) significantly boosts the first-lactation production of dairy cows, with no apparent plateau in this relationship [1, 2]. Specifically, an increase in preweaning ADG has demonstrated a substantial improvement in milk yield, energy-corrected milk, and fat-corrected milk during the first lactation of primiparous heifers, resulting in approximate increases of 829 kg, 754 kg, and 763 kg, respectively [3]. Additional published studies have further confirmed that higher preweaning growth in heifers positively links to both first lactation milk yield and fertility [3, 4]. Together, a higher preweaning ADG of calves is beneficial for improving adult lactation performance, thereby meeting the growing demand for high-quality protein.

The rumen serves as the primary organ for the digestion and fermentation of feed by rumen microbes, resulting in the production of volatile fatty acids (VFAs) as the main fermentation end products [5]. Following this fermentation process, nutrients, including VFAs and minerals, are absorbed through the rumen epithelial wall and utilized by the animals [5]. More than 70% of ruminal VFAs are absorbed through the rumen epithelium, providing a valuable energy resource [6]. Rumen bacteria not only function as an exceptional source of high-quality protein flowing into the small intestine for digestion but also significantly influence the host phenotype, including metrics such as ADG [7]. Paz et al. [8] reported that rumen bacteria of heifer have been found to explain 25.3% of the variation in ADG. Research by Xie et al. [9] underscored the importance of specific species of rumen *Lachnospiraceae* involved in carbohydrate metabolism, contributing to variations in residual feed intake. This highlights the potential for manipulating ruminal microbes or their functions to enhance the feed efficiency of dairy cows [9]. The rumen metabolome offers a valuable approach to explore ruminal metabolism [10], where microbial metabolites play a more substantial role in shaping the host phenotype than taxonomic composition [11]. Integrating metagenome and metabolome studies suggested that rumen microbial variations impact ruminal carbohydrate and protein metabolism [12]. This improved the production of ruminal propionate, butyrate, and amino acids in high ADG goats, thereby enhancing energy and nutrient availability for goats [12]. Consequently, employing multi-omics methods is instrumental in understanding how the ruminal microbial characteristics influence rumen metabolism, subsequently leading to changes in nutrients and energy that can be utilized by the host, ultimately resulting in variations in preweaning ADG in calves.

During the preweaning period, when the calf’s rumen is not yet fully developed, hindgut microbial fermentation plays a relatively important role in providing nutrition and energy for the host [13]. Undigested dietary components transit to the hindgut, where microbial metabolism produces crucial compounds that play a pivotal role in regulating the growth and development of preweaning calves [13, 14]. Kodithuwakku et al. [15] suggested that an optimized hindgut environment, characterized by a higher abundance of probiotics, can enhance calf growth during the early stages of life. Elolimy et al. [16] demonstrated the association between the hindgut microbiome and metabolome with the variation in residual feed intake of preweaning calves, emphasizing their significance. Therefore, understanding how hindgut microbes in preweaning calves regulate ADG through hindgut metabolism is of paramount importance. Additionally, a published study highlighted the correlation between altered plasma metabolome and variations in the ADG of beef steers, underscoring the contribution of host metabolism to the host phenotype [17]. Recent studies have also indicated potential interactions between the host metabolome and both the hindgut and rumen microbiomes [11, 18]. Thus, rumen and hindgut microbes not only individually affect rumen and hindgut metabolism but also might collectively influence host metabolism, thereby resulting in variations in ADG of preweaning calves.

In this study, we hypothesized that preweaning calves, under the influence of gastrointestinal microbes, exhibit variations in rumen, hindgut, and plasma metabolism, leading to changes in nutrient and energy levels and ultimately impacting the preweaning ADG. To test this hypothesis, our research aimed to investigate the rumen metagenome, rumen metabolism, hindgut metagenome, hindgut metabolism, plasma metabolism, rumen fermentation indicators, and growth parameters in preweaning calves with different ADG. Additionally, extensive in vitro cultures were performed to isolate gastrointestinal microbes to further validate our hypothesis. The goal of this study is to investigate the mechanisms by which gastrointestinal microbes regulate ADG in preweaning calves and to provide strain resources for the validation of these mechanisms and for the subsequent regulation of ADG in preweaned calves by modulating gastrointestinal microbes.

## Materials and methods

### Animal management, diet, and experimental design

This study complied with the requirements of the Animal Ethics Committee of the Chinese Academy of Agricultural Sciences (Beijing, China). A total of 106 newborn Holstein heifer calves were obtained from a commercial dairy farm in Beijing, China. These calves were individually housed in hutches until they reached 60 days old. Newborn calves received 4 L of colostrum within 2 h after birth and 2 L of colostrum after 8 h. Colostrum was also provided to all calves at 0800, 1500, and 2100 h with a total of 6 L/day for 2 to 3 days old. Milk replacer (Table S1) was provided to all calves at 0800, 1500, and 2100 h with a total of 7.5 L/day for 4 to 9 days old, 9 L/day for 10 to 14 days old, 10.5 L/day for 15 to 29 days old, and 12 L/day for 30 to 60 days old. All calves were weaned at 60 days old. All calves were allowed unrestricted access to starter and water starting from the 5 days old (Table S1).

The birth weight and body weight at 60 days old were recorded in young calves. The difference between body weight at 60 days old and the birth weight was divided by the age of the day to obtain the ADG. Detailed documentation of the initial quantity of milk replacer provided, the remaining amount, and the consumption of starter feed was maintained to calculate the average daily feed intake (ADFI). The calves were systematically grouped based on their preweaning ADGs: those with higher preweaning ADGs were categorized into the higher ADG group (HADG; *n* = 8), while those with comparatively lower preweaning ADGs were classified into the lower ADG group (LADG, *n* = 8). Throughout the grouping process, it was ensured that birth weight and ADFI were no significantly different between the two groups. This meticulous approach aimed to mitigate any potential influence stemming from variations in nutrition and birth weight on ADG outcomes. It is noteworthy that both groups of calves shared a common paternal lineage, a deliberate choice made to minimize potential confounding effects arising from genetic factors.

### Sample collection and measurement

Blood samples (10 mL) were collected from each 60-day-old calf through the jugular vein using vacuum blood collection tubes containing anticoagulant, obtained before the morning feeding. These samples were subsequently centrifuged at 2000 g for 10 min to isolate plasma, which was then stored in tubes and frozen at − 80 °C for metabolite extraction. Ruminal contents were obtained from the 60-day-old calves using an oral stomach tube before the morning feeding. The collected ruminal contents were divided into three tubes and stored at − 80 °C for subsequent microbial DNA extraction, metabolite extraction, and analysis of volatile fatty acids (VFAs) concentration using gas chromatography [19]. On the same day, fresh fecal samples were gathered from the calves using rectal finger stimulation with a sterile-gloved hand before the morning feed. These fecal samples were collected into two tubes and immediately frozen at − 80 °C for microbial DNA extraction and metabolite extraction.

### DNA extraction, metagenome sequencing, and sequence analysis

Total genomic DNA was individually extracted from both ruminal contents and fecal samples using the FastDNA SPIN for Soil Kit (MP Biomedicals, Solon, USA) following the manufacturer’s protocol. The concentration and purity of the extracted DNA were assessed using the TBS-380 spectrophotometer and NanoDrop 2000, respectively. Additionally, DNA quality was evaluated using 1% agarose gel electrophoresis. Subsequently, the extracted DNA underwent fragmentation using the Covaris M220 (Gene Company Limited, China) to achieve an average size of approximately 400 bp, enabling the preparation of paired-end libraries. The construction of paired-end libraries was carried out using the NEXTFLEX® Rapid DNA-Seq kit (Bioo Scientific, Austin, TX, USA), where adapters containing sequencing primer hybridization sites were ligated to the blunt-end fragments. Paired-end sequencing was conducted on the Illumina NovaSeq platform (Illumina Inc., San Diego, CA, USA) at Majorbio Bio-Pharm Technology Co., Ltd. (Shanghai, China).

Upon sequencing, raw reads were processed to obtain high-quality filtered reads for subsequent analyses. This involved adaptor trimming and removal of low-quality reads (length < 50 bp, quality value < 20, or containing N bases) using fastp (https://github.com/OpenGene/fastp, version 0.20.0) [20]. To eliminate any potential host contamination, the reads were aligned to the bovine genome (bosTau8 3.7, 10.18129/B9.bioc.BSgenome.Btaurus.UCSC.bosTau8) using BWA (http://bio-bwa.sourceforge.net, version 0.7.9a) [21]. Metagenomics data were assembled using MEGAHIT (https://github.com/voutcn/megahit, version 1.1.2), which makes use of succinct *de* Bruijn graphs [22]. Then, contigs with a length ≥ 300 bp were deemed as the conclusive assembly outcome, subsequently utilized for subsequent gene prediction and annotation analyses. Open reading frames (ORFs) within each assembled contig were predicted utilizing MetaGene (http://metagene.cb.k.u-tokyo.ac.jp/) [23]. ORFs predicted with a length ≥ 100 bp were extracted, and their translation into amino acid sequences was performed using the NCBI translation table (http://www.ncbi.nlm.nih.gov/Taxonomy/taxonomyhome.html/index.cgi?chapter=tgencodes#SG1). A nonredundant gene catalog was constructed using CD-HIT (http://www.bioinformatics.org/cd-hit/, version 4.6.1) [24] with 90% sequence identity and 90% coverage. High-quality reads were aligned to the nonredundant gene catalogs to calculate gene abundance with 95% identity using SOAPaligner (http://soap.genomics.org.cn/, version 2.21) [25].

### Taxonomic and functional annotation

Representative sequences of the nonredundant gene catalog were performed using Diamond [26] (http://www.diamondsearch.org/index.php, version 0.8.35) against the NR database with an *e*-value cutoff of 1e-5 for taxonomic annotations. The Kyoto Encyclopedia of Genes and Genomes (KEGG) annotation was performed by utilizing Diamond to search against the KEGG database (http://www.genome.jp/kegg/) with an *e*-value threshold of 1e-5 [27]. The carbohydrate-active enzymes (CAZy) annotation was performed using hmmscan (http://hmmer.janelia.org/search/hmmscan) [28]. The relative abundance of a taxon/function in a subject was calculated by summing the transcripts per million (TPM) values assigned to that taxon/function and then by dividing the TPM by the total TPM of the subject. Only taxa and functions with a relative abundance > 0.01% in at least 50% of calves were used for downstream analysis.

### Analysis of rumen, feces, and plasma metabolome

The LC–MS/MS analyses of rumen metabolome [29], feces metabolome [30], and plasma metabolome [31] were conducted on Thermo UHPLC-Q Exactive HF-X system at Majorbio Bio-Pharm Technology Co., Ltd. (Shanghai, China). The data processing was carried out as previously reported [31]. The LC/MS raw data was preprocessed using Progenesis QI software (Waters Corporation, Milford, USA). At the same time, the search and identification of metabolites were conducted using the Human Metabolome Database (HMDB, http://www.hmdb.ca/), Kyoto Encyclopedia of Genes and Genomes Database (KEGG, https://www.genome.jp/kegg/), METLIN (https://metlin.scripps.edu/), and the Majorbio database.

### In vitro isolation of rumen microbes

Rumen contents from preweaning calves at the same commercial dairy farm were collected to isolate ADG-associated rumen microbes. The fresh rumen contents were diluted separately in sterile PBS (phosphate-buffered saline) at 10^3^, 10^4^, and 10^5^-fold dilutions for plating onto PYG (peptone yeast extract glucose) solid culture medium (Qingdao Hope Biotechnology Co. Ltd., Shandong, China) (Table S2). All procedures were executed within the Defendor AMW 500, an anaerobic and microaerophilic workstation (Huayue Inc., Guangdong, China). The workstation maintained specific parameters: a temperature of 37 °C, oxygen concentration set at 0%, carbon dioxide concentration at 3.8%, nitrogen concentration at 96.2%, and humidity sustained at 74% RH. After cultivation for 48 h in the anaerobic and microaerophilic workstation, individual colonies were selected and streaked for purification for at least three generations to acquire the final screened strains. These final strains were subsequently identified using 16S rRNA sequencing at RuiBiotech Co., Ltd., Beijing, China. If these final strains belong to ADG-associated rumen microbes, further analysis will be conducted.

The identified strain (we named *Acidaminococcus fermentans* P41) was subsequently subjected to whole genome sequencing at RuiBiotech Co., Ltd., Beijing, China. Genomic DNA of *A. fermentans* P41 was extracted using magnetic soil and stool DNA kit according to manufacturer’s protocol. Purified genomic DNA was quantified, and high-quality DNA was used to do further research. The draft genome sequence analysis was carried out using the Illumina HiSeq platform (Illumina Inc., San Diego, CA, USA). All subsequent bioinformatics analyses were performed using the online platform of Majorbio Cloud Platform (www.majorbio.com) from Majorbio Bio-Pharm Technology Co., Ltd. (Shanghai, China). Sequencing data underwent filtering via fastp software (v0.19.6) [20] before assembly using SOPA de novo version 2.04, both using default parameters [32]. Glimmer [33] was employed for coding sequence (CDS) prediction, tRNA-scan-SE [34] for tRNA prediction, and Barrnap for rRNA prediction, all employing default parameters. Predicted CDSs were annotated from Clusters of Orthologous Groups of proteins (COG) and KEGG databases through sequence alignment tools such as BLASTP, Diamond, and HMMER, with default parameters. In brief, query protein sets were aligned with databases, retrieving annotations from best-matched subjects (*e*-value < 10^−5^) for gene annotation.

### Metabolic characterization of *A. fermentans* P41

The *A. fermentans P41* was cultured in PYG liquid medium for 48 h, followed by acid production detection and untargeted metabolomic analysis. The concentration of volatile fatty acids (VFAs) was measured using gas chromatography [19]. LC–MS analyses were conducted using a Vanquish UHPLC system (Thermo Fisher Scientific, USA) at PANOMIX Biomedical Tech Co., Ltd. (Suzhou, China). Raw data were converted to mzXML format using MSConvert in ProteoWizard software (v3.0.8789) [35]. Subsequent data processing involved feature detection, retention time correction, and alignment using the “XCMS” package (v3.12.0) in R [36]. Metabolites were identified by matching accurate mass and MS/MS data with various databases, including HMDB (http://www.hmdb.ca), MassBank (http://www.massbank.jp/), KEGG (https://www.genome.jp/kegg/), LIPID MAPS (http://www.lipidmaps.org), mzCloud (https://www.mzcloud.org), and a proprietary metabolite database constructed by PANOMIX Biomedical Tech Co., Ltd.

### In vitro fermentation experiment

Four Holstein cows with rumen fistulae served as donors of rumen fluid. Rumen fluid was collected at 9:00 AM, post-milking and pre-feeding, filtered through four layers of gauze, thoroughly mixed, and stored in a thermos. An in vitro fermentation system was used to collect the fermentation fluid. Each bottle contained 200 mL of Menke medium, 100 mL of rumen fluid, and 10 g of substrate. Three types of substrates were used: 10 g of milk replacer (Table S1), 10 g of starter (Table S1), and a mix of 5.79 g of milk replacer and 4.21 g of starter (Table S1). Each substrate had PYG liquid medium added, serving as the control group (CON), and PYG medium inoculated with *A. fermentans P41*, serving as the treatment group (AFP). The addition amount of *A. fermentans P41* is 10^7^ CFU/mL. The headspace of the fermentation bottles was purged with carbon dioxide. Afterward, the fermentation bottles were placed under conditions at 39 °C for 48 h. Following fermentation, the fermentation fluid was collected for VFA determination. For each fermentation sample of different treatments, three replicates were set up.

### Statistical analysis

Statistical analyses of ADFI, ADG, birth weight, rumen fermentation parameters, and in vitro fermentation parameters were performed using the independent sample *T*-test in IBM SPSS Statistics 25 software, with a significance level of *P* < 0.05. The microbial alpha diversity between the two groups was evaluated utilizing the Wilcoxon rank-sum test, with a false discovery rate (FDR) adjusted *P*-value < 0.05 signifying significance. To assess beta diversity, the principal component analysis (PCA) was conducted employing the Bray–Curtis distance algorithm, with intergroup differences assessed through the Adonis method. Microbial domains were compared using Wilcoxon rank-sum test, with the FDR adjusted *P*-value < 0.05 being considered as significantly different. Microbial phyla, genera, and species were compared using linear discriminant analysis effect size (LEfSe), with LDA score > 2 and *P*-value < 0.05 being considered as significantly different. The relative abundances of KEGG pathways, KEGG modules, KEGG enzymes, and CAZymes were also compared between two groups using LEfSe, and significant differences were considered by an LDA score > 2 and *P*-value < 0.05. Spearman’s rank correlation was used for all correlation analysis in this study (Spearman’s |r| > 0.50 and *P* < 0.05). Multiplex networks were visualized using Gephi software (version 0.10).

Regarding the statistical analysis of metabolites, the R package “ropls” (Version 1.6.2) was used to perform orthogonal least partial squares discriminant analysis (OPLS-DA) and seven-cycle interactive validation evaluating the stability of the model. The metabolites with VIP > 1 and *P* < 0.05 were determined as significantly different metabolites based on the variable importance in the projection (VIP) obtained by the OPLS-DA model and the *P*-value generated by Student’s *t*-test. Further, enrichment analysis was conducted using the online platform MetaboAnalyst 5.0 (https://www.metaboanalyst.ca/) [37].

## Results

### Preweaning calves had differential growth rates and rumen fermentation

**Fig. 1:**
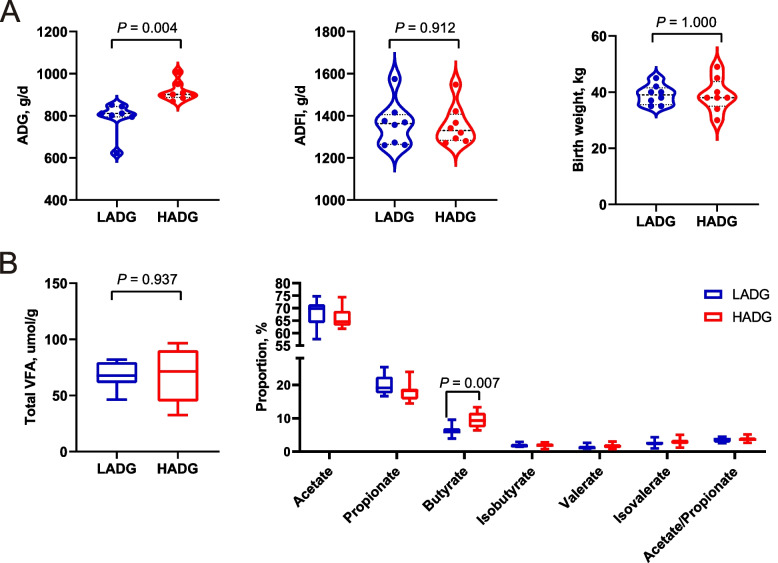
Comparison of phenotypes between HADG and LADG calves. **A** Differences in average daily gain (ADG), average daily feed intake (ADFI), and birth weight between HADG and LADG calves. **B** Differences in rumen fermentation between HADG and LADG calves. HADG, higher average daily gain group; LADG, lower average daily gain group

The HADG group displayed notably elevated ADG in calves compared to the LADG group (*P* < 0.05; Fig. 1A, Table S3). Intriguingly, the proportion of butyrate in the rumen of HADG calves significantly surpassed that in LADG calves (*P* < 0.05; Fig. 1B, Table S3). This observation may suggest that different rumen microbes drive variations in rumen fermentation parameters, underscoring the imperative need for further exploration of the rumen microbiome.

### Compositional profiles of the rumen microbiome and taxonomic differences between the HADG and LADG calves

Rumen metagenome sequencing generated a total of 1,489,259,720 reads with 93,078,733 ± 1,813,950 (mean ± standard error of the mean) reads per sample. After quality control and removing host genes, a total of 1,251,023,610 reads were reserved with 78,188,976 ± 2,843,887 reads per sample. After contig assembly, a total of 5,372,043 contigs were generated, with 335,753 ± 30,251 contigs per sample (Table S4).

**Fig. 2:**
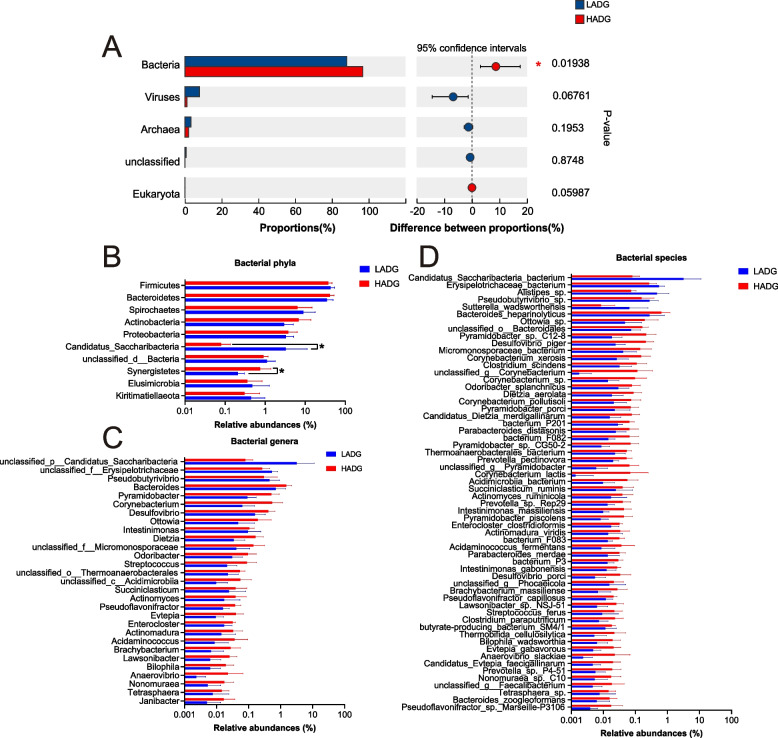
Comparison of rumen microbial taxa between HADG and LADG calves. **A** Comparison of rumen microbial domains between HADG and LADG calves. Significantly different domains were tested by Wilcoxon rank-sum test with adjusted *P*-value of < 0.05. * Means adjusted *P* < 0.05. **B** Top 10 rumen bacterial phyla. * Means significantly different phyla. **C** All significantly different rumen bacterial genera. **D** All significantly different rumen bacterial species. Microbial phyla, genera, and species were compared using linear discriminant analysis effect size (LEfSe), with LDA score > 2 and *P*-value < 0.05 being considered as significantly different. HADG, higher average daily gain group; LADG, lower average daily gain group

Alpha-diversity calculations revealed no divergence of the Shannon and Chao indices (adjusted *P* > 0.05; Fig. S1A), indicating unchanged rumen microbial richness and evenness between the two groups. The distribution of microbial domains within the rumen microbiome of the 16 calves includes bacteria (92.41%), viruses (4.56%), archaea (2.66%), and eukaryotes (0.03%) (Table S5). The Wilcoxon rank-sum test revealed a significant enrichment in the relative abundance of bacteria (96.75%) in the rumen microbiome of HADG calves compared to LADG calves (88.07%) (adjusted *P* < 0.05) (Fig. 2A, Table S5). The PCA showed clear separation between the HADG and LADG calves based on bacterial species level (*P* < 0.05; Fig. S1B). Consequently, only bacteria were considered for downstream comparison of rumen microbial taxa between the calf groups. The predominant bacterial phyla within the rumen microbiome of the 16 calves were Firmicutes (40.34%), Bacteroidetes (38.24%), and Spirochaetes (7.71%); the dominant bacterial genera were *Prevotella* (15.54%), *unclassified_c__Clostridia* (10.18%), *unclassified_o__Bacteroidales (10.06*%), *unclassified_f__Lachnospiraceae* (6.92%), and *Treponema* (6.66%); and the dominant bacterial species included *Prevotella* sp. (12.66%), Clostridia bacterium (10.18%), Bacteroidales bacterium (9.94%), Lachnospiraceae bacterium (6.79%), and Bacteroidaceae bacterium (4.78%) (Table S6). LEfSe analyses were conducted to assess differences at the bacterial phylum, genus, and species levels. At the phylum level, Synergistetes were significantly higher in the rumen microbiome of HADG calves, whereas *Candidatus* Saccharibacteria were notably higher in the rumen microbiome of LADG calves (LDA > 2, *P* < 0.05; Fig. 2B, Table S7). Remarkably, the rumen microbiome of HADG calves exhibited enrichment in the majority of significantly different bacterial genera or species compared to LADG calves (Table S7). Specifically, 29 significantly different genera were detected, with 26 genera enriched in the rumen microbiome of HADG calves and only 3 in the rumen microbiome of LADG calves (LDA > 2, *P* < 0.05; Fig. 2C). Likewise, at the species level, 55 species showed significantly higher relative abundances in the rumen microbiome of HADG calves (HADG-enriched rumen microbes), whereas only five species exhibited significant enrichment in the rumen microbiome of LADG calves (LADG-enriched rumen microbes) (LDA > 2, *P* < 0.05; Fig. 2D). The dominance of HADG calves in rumen bacterial composition suggests potentially more active metabolic capabilities within the rumen microbes of HADG calves.

Correlations between rumen bacterial species and host phenotypes (proportion of butyrate and ADG) were identified. Seventeen HADG-enriched rumen microbes, including *Acidaminococcus fermentans, bacterium F082, bacterium P201, Candidatus* Evtepia faecigallinarum, *Desulfovibrio piger*, *Enterocloster clostridioformis*, *Evtepia gabavorous*, *Intestinimonas gabonensis*, *Prevotella pectinovora*, *Prevotella* sp. *Rep29*, *Pseudoflavonifractor capillosus*, *Pyramidobacter piscolens*, *Pyramidobacter porci*, *Pyramidobacter* sp. C12-8, *Pyramidobacter* sp. CG50-2, *unclassified_g_Faecalibacterium*, and *unclassified_g_Pyramidobacter*, displayed positive correlations with both ADG and the proportion of butyrate. These findings categorized them as ADG-associated rumen microbes (*R* > 0.50, *P* < 0.05; Fig. S2).

### Functional profiles of the rumen microbiome and differential functions between the HADG and LADG calves

**Fig. 3:**
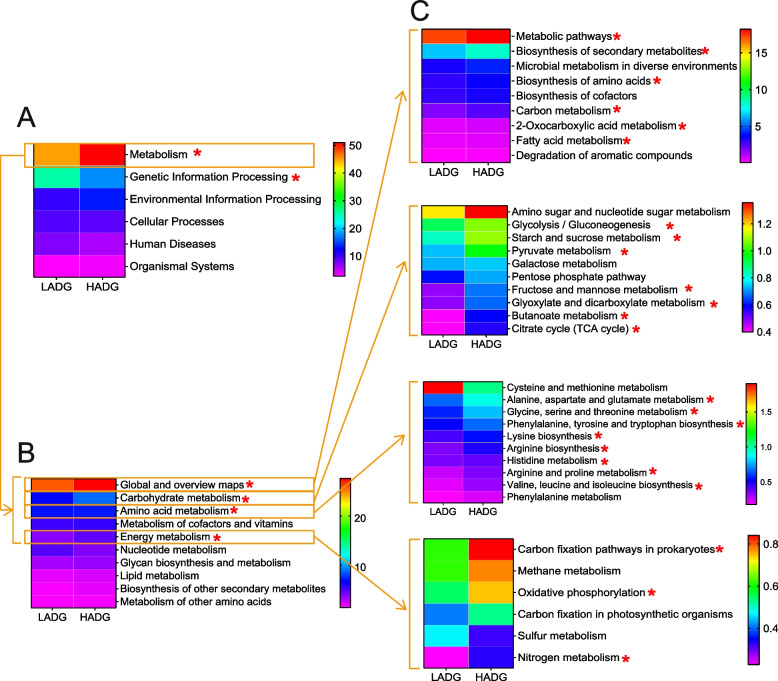
Difference in functional capacities of the rumen microbiome between HADG and LADG calves. Relative abundance of function KEGG pathway **A** level 1, **B** level 2 (top 10 of metabolism), and **C** level 3 (top 9 of global and overview maps, top 10 of carbohydrate metabolism, top 10 of amino acid metabolism, and top 6 of energy metabolism). KEGG pathways were compared using linear discriminant analysis effect size (LEfSe), with LDA score > 2 and *P*-value < 0.05 being considered as significantly different. * Means LDA score > 2 and *P*-value < 0.05. HADG, higher average daily gain group; LADG, lower average daily gain group

**Fig. 4:**
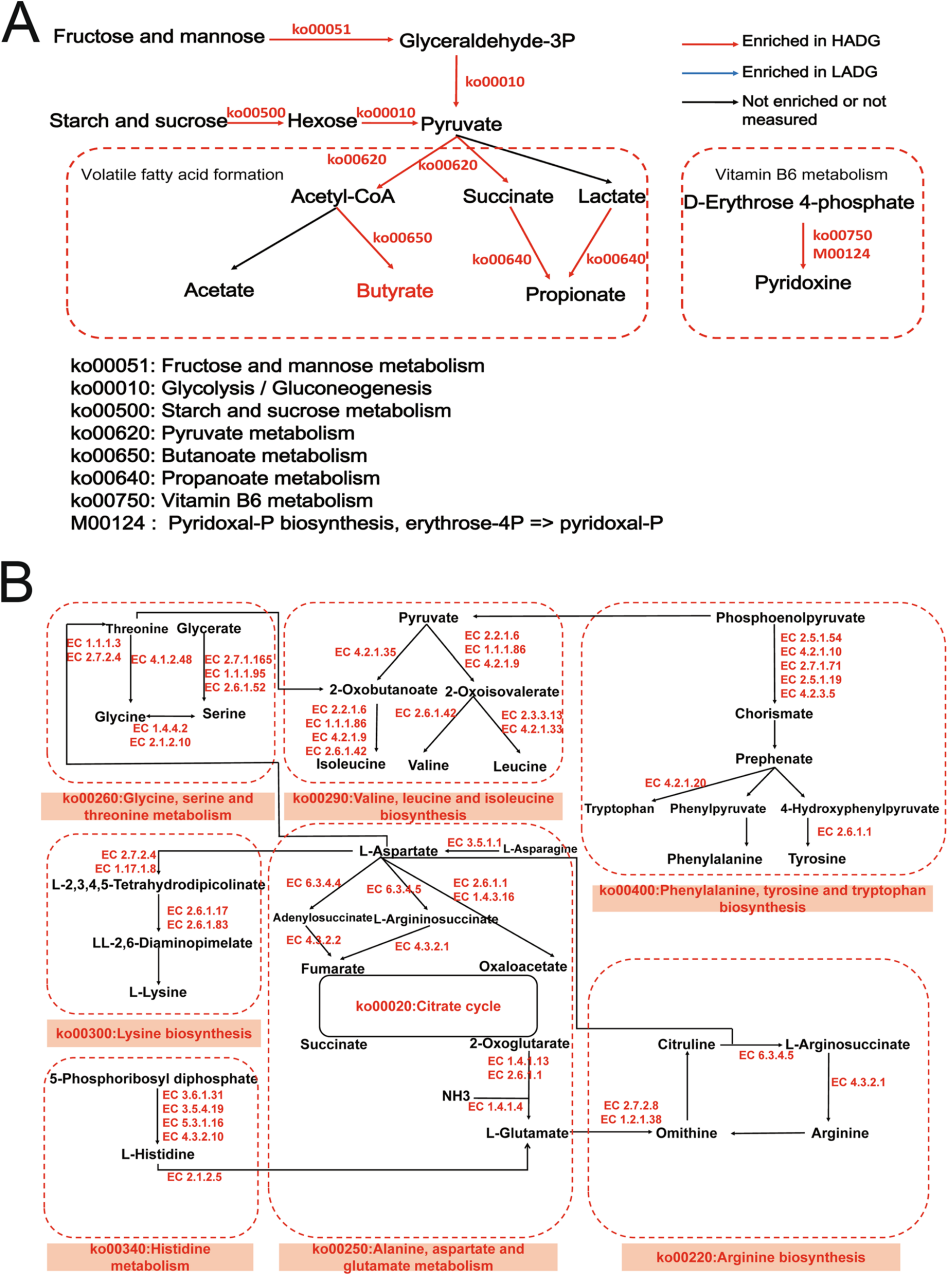
Microbial functions involved in carbohydrate metabolism, amino acid metabolism, and vitamin metabolism in the rumen of HADG and LADG calves. **A** Metabolic pathways involved in VFAs biosynthesis. **B** Amino acid metabolism pathways. The red text represents KEGG pathways, KEGG modules, KEGG enzymes, or metabolites enriched in the rumen microbiome of HADG calves (LDA score > 2, *P*-value < 0.05). HADG, higher average daily gain group; LADG, lower average daily gain group

Consistent with the observed microbiome composition, the rumen microbiome of HADG calves also demonstrates functional dominance. We conducted functional analysis utilizing Kyoto Encyclopedia of Genes and Genomes (KEGG) profiles and carbohydrate-active enzymes (CAZymes) profiles. The PCA showed clear separation between the HADG and LADG calves based on KEGG level-3 pathways (*P* < 0.05; Fig. S3A). Specifically, analysis of KEGG level-1 pathways identified predominant categories within the rumen microbiome of 16 calves: “metabolism” (47.97%), “genetic information processing” (21.55%), “environmental information processing” (11.78%), “cellular processes” (9.11%), “human diseases” (6.65%), and “organismal systems” (2.95%) (Table S8). Notably, “metabolism” exhibited significant enrichment in the rumen microbiome of HADG calves (51.00%) compared to LADG calves (44.93%), while “genetic information processing” showed enrichment in the rumen microbiome of LADG calves (25.36%) compared to HADG calves (17.73%) (LDA > 2, *P* < 0.05; Fig. 3A, Table S8). At KEGG level-2, within the “metabolism” pathway, subcategories such as “global and overview maps,” “carbohydrate metabolism,” “amino acid metabolism,” and “energy metabolism” were significantly enriched in the rumen microbiome of HADG calves (LDA > 2, *P* < 0.05; Fig. 3B, Fig. S4A). Further exploration at KEGG level-3 identified 60 significantly different pathways, with 52 enriched in the rumen microbiome of HADG calves (LDA > 2, *P* < 0.05; Fig. 3C, Fig. S4B). Specifically, the six “carbohydrate metabolism” pathways (fructose and mannose metabolism, glycolysis/gluconeogenesis, starch and sucrose metabolism, pyruvate metabolism, butanoate metabolism, and propanoate metabolism), seven “amino acids metabolism” pathways (arginine biosynthesis; histidine metabolism; phenylalanine, tyrosine, and tryptophan biosynthesis; glycine, serine, and threonine metabolism; valine, leucine, and isoleucine biosynthesis; lysine biosynthesis; and alanine, aspartate, and glutamate metabolism), six “global and overview maps” pathways (metabolic pathways, biosynthesis of secondary metabolites, biosynthesis of amino acids, carbon metabolism, 2-oxocarboxylic and metabolism, and fatty acid metabolism), three “energy metabolism” pathways (carbon fixation pathways in prokaryotes, oxidative phosphorylation, and nitrogen metabolism), and one “metabolism of cofactors and vitamins” pathway (“vitamin B6 metabolism”) were all HADG-enriched pathways (LDA > 2, *P* < 0.05; Fig. 3C, Fig. S4B). Moreover, the comparison of KEGG modules unveiled 46 HADG-enriched and two LADG-enriched modules (LDA > 2, *P* < 0.05; Fig. S5). Notably, within vitamin metabolism, only the “pyridoxal-P biosynthesis, erythrose-4P =  > pyridoxal-P” module (M00124) showed enrichment in the rumen microbiome of HADG calves (LDA > 2, *P* < 0.05; Fig. S5). For a comprehensive understanding of carbohydrate, amino acid, and vitamin metabolism, key pathways and modules were integrated for further analysis (Fig. 4A, B). Examining KEGG enzymes involved in amino acid metabolism revealed significant enrichment in amino acid biosynthesis enzymes within the rumen microbiome of HADG calves (LDA > 2, *P* < 0.05; Fig. 4B; Fig. S6).

The PCA showed no separation between the HADG and LADG calves based on family-level CAZymes (*P* > 0.05; Fig. S3B). In terms of CAZyme profiles within the rumen microbiome of 16 calves, the class-level CAZymes, including glycoside hydrolases (GHs, 42.42%), glycosyl transferases (GTs, 36.29%), carbohydrate esterases (CEs, 14.02%), auxiliary activities (AAs, 3.24%), carbohydrate-binding modules (CBMs, 2.81%), polysaccharide lyases (PLs, 1.11%), and cellulosome modules (CMs, 1.11%), showed that CEs and PLs were enriched in the rumen microbiome of HADG calves (LDA > 2, *P* < 0.05; Fig. S7A). We further compared the relative abundance of family-level CAZymes, and 70 significantly different CAZymes were identified between the two groups. Only 4 CAZymes (1 CBM, 1 GH, and 2 GTs) were enriched in the rumen microbiome of LADG calves, while 66 CAZymes (4 CEs, 55 GHs, 5 GTs, and 2 PLs) were enriched in the rumen microbiome of HADG calves (LDA > 2, *P* < 0.05; Fig. S7B). This elucidates the advantage of the rumen microbiome of HADG calves in carbohydrate metabolism.

### Relationships between phenotypes, rumen microbial species, and rumen microbial functions

We conducted Spearman’s rank correlation analysis to explore the interrelationships among ADG-associated rumen microbes, KEGG pathways related to carbohydrate metabolism, the proportion of butyrate, and ADG. Our multiplex network revealed significant positive correlations between 17 ADG-associated rumen microbes and 6 KEGG pathways linked to carbohydrate metabolism (*R* > 0.50, *P* < 0.05; Fig. S8). Moreover, these six KEGG pathways demonstrated positive correlations with the proportion of butyrate, which, in turn, displayed a positive relationship with ADG (*R* > 0.50, *P* < 0.05; Fig. S8).

Similarly, we performed Spearman’s rank correlation analysis to assess the connections between ADG-associated rumen microbes, KEGG pathways associated with amino acid metabolism, and ADG. Our findings indicated that the 17 ADG-associated rumen microbes were positively correlated with the eight KEGG pathways related to amino acid metabolism (*R* > 0.50, *P* < 0.05; Fig. S9). Notably, these eight pathways also showed positive correlations with ADG (*R* > 0.50, *P* < 0.05; Fig. S9). In sum, the 17 ADG-associated rumen microbes may be involved in ruminal carbohydrate and amino acid metabolism, potentially promoting calf growth.

### Rumen metabolome profiling differed between higher and lower performance calves

**Fig. 5:**
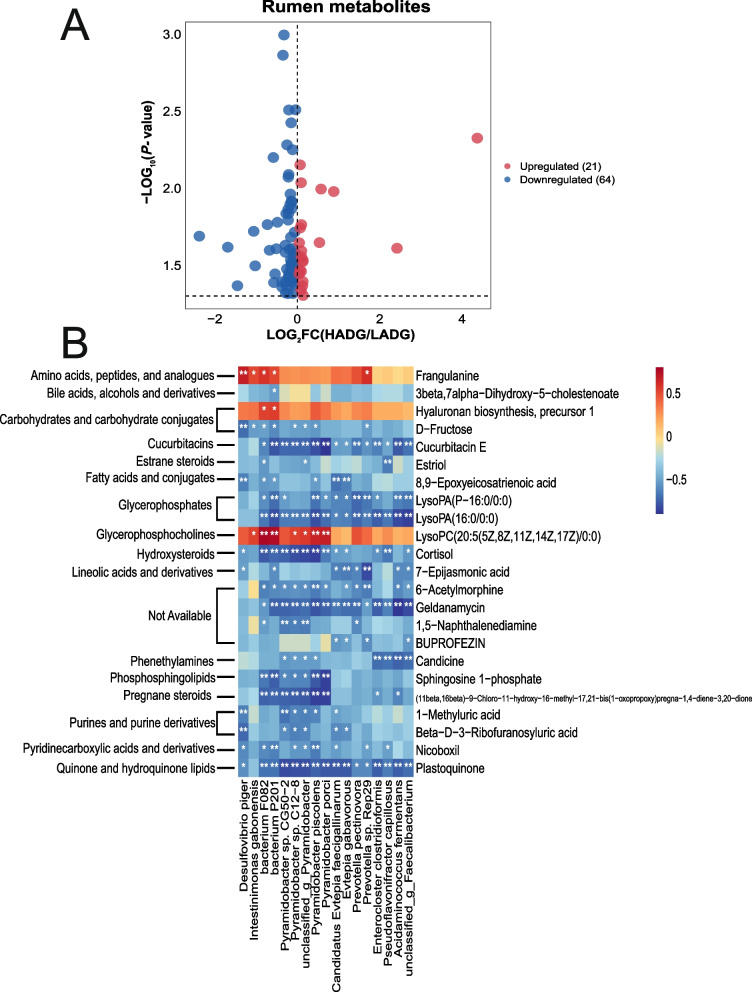
Rumen metabolites and their relationship with rumen microbes. **A** Volcano plot of ruminal differential metabolites (VIP > 1, *P* < 0.05). **B** Correlations between the ADG-associated rumen metabolites and the ADG-associated rumen microbes (Spearman’s correlation). * Means Spearman’s |r| > 0.50 and *P* < 0.05, ** means Spearman’s |r| > 0.50 and *P* < 0.01. HADG, higher average daily gain group; LADG, lower average daily gain group

The comparison analysis using *t*-tests and variable importance in projection (VIP) identified 85 significantly different rumen metabolites between the two groups (*P* < 0.05, VIP > 1; Fig. 5A, Table S9). Among these, the levels of 21 metabolites increased, while 64 metabolites decreased in the rumen of HADG calves compared to LADG calves (*P* < 0.05, VIP > 1; Fig. 5A). A correlation analysis was conducted between these varied metabolites and ADG using Spearman’s rank correlation. This analysis revealed that 23 metabolites exhibited significant correlations with ADG, termed as ADG-associated rumen metabolites (*R* > |0.50|, *P* < 0.05; Table S10). Notably, three metabolites were significantly higher in HADG calves and positively associated with ADG: LysoPC(20:5(5Z, 8Z, 11Z, 14Z, 17Z)/0:0), hyaluronan biosynthesis precursor 1, and frangulanine (HADG-associated rumen metabolites) (Table S9; Table S10). Conversely, 20 metabolites, including D-fructose, sphingosine 1-phosphate, and 8,9-epoxyeicosatrienoic acid, were significantly higher in LADG calves and exhibited negative associations with ADG (LADG-associated rumen metabolites) (Table S9; Table S10).

Upon further analysis between ADG-associated rumen microbes and ADG-associated rumen metabolites, several HADG-enriched rumen microbes, such as *bacterium P201*, *bacterium F082*, *P. piscolens*, *P.* sp. C12-8, and *unclassified_g_Pyramidobacter*, exhibited negative correlations with D-fructose and sphingosine 1-phosphate (*R* <  − 0.50, *P* < 0.05; Fig. 5B). Moreover, *P. piscolens*, *bacterium P201*, and *bacterium F082* showed negative associations with 8,9-epoxyeicosatrienoic acid (*R* <  − 0.50, *P* < 0.05; Fig. 5B). Interestingly, *bacterium P201* and *bacterium F082* displayed positive relationships with HADG-associated rumen metabolites, including hyaluronan biosynthesis precursor 1, LysoPC(20:5(5Z, 8Z, 11Z, 14Z, 17Z)/0:0) and frangulanine (*R* > 0.50, *P* < 0.05; Fig. 5B). Additionally, four *Pyramidobacter* species (*P.* sp. C12 − 8, *unclassified_g_Pyramidobacter*, *P. piscolens*, and *P. porci*) exhibited positive correlations with LysoPC(20:5(5Z, 8Z, 11Z, 14Z, 17Z)/0:0) (*R* > 0.50, *P* < 0.05; Fig. 5B).

### Compositional profiles of the fecal microbiome and taxonomic differences between the HADG and LADG calves

Fecal metagenome sequencing generated a total of 1,503,357,586 reads with 93,959,849 ± 2,650,883 reads per sample. After quality control and removing host genes, a total of 1,261,138,580 reads were reserved with 78,821,161 ± 3,006,992 reads per sample. After contig assembly, a total of 2,370,226 contigs were generated, with 148,139 ± 16,939 contigs per sample (Table S11).

**Fig. 6:**
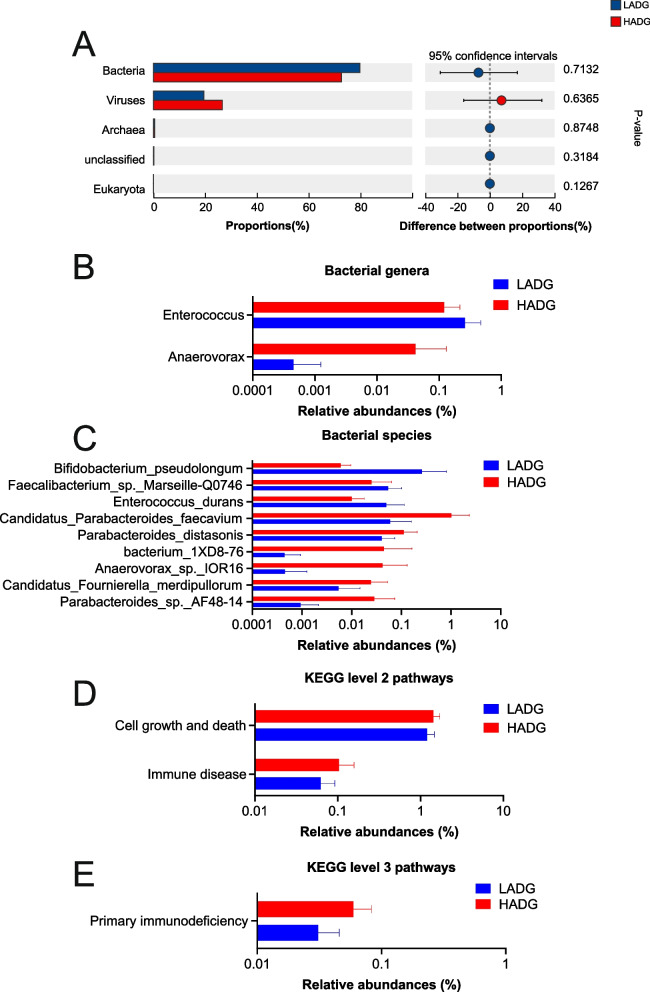
Fecal microbiome taxonomic and functional features. **A** Comparison of fecal microbial domains between HADG and LADG calves. Significantly different domains were tested by Wilcoxon rank-sum test with adjusted *P*-value of < 0.05. * Means adjusted *P* < 0.05. **B** All significantly different fecal bacterial genera. **C** All significantly different fecal bacterial species. **D** All significantly different KEGG level 2 pathways. **E** All significantly different KEGG level 3 pathways. Microbial genera, species, and KEGG pathways were compared using linear discriminant analysis effect size (LEfSe), with LDA score > 2 and *P*-value < 0.05 being considered as significantly different. HADG, higher average daily gain group; LADG, lower average daily gain group

Alpha-diversity calculations revealed no divergence of the Shannon and Chao indices (adjusted *P* > 0.05; Fig. S10A), indicating unchanged fecal microbial richness and evenness between the two groups. The microbial domains present in the fecal microbiome of 16 calves comprised bacteria (76.27%), viruses (23.06%), archaea (0.47%), and eukaryotes (0.02%). However, no significant difference was observed between the two groups (Fig. 6A; Table S12). Given the prevalence of bacteria in feces, our analysis focused on this microbial domain for downstream investigations. The PCA showed no separation between the HADG and LADG calves based on bacterial species (*P* > 0.05; Fig. S10B). The predominant bacterial phyla within the fecal microbiome of 16 calves were Firmicutes (59.24%), Bacteroidetes (27.07%), Actinobacteria (10.40%), and Proteobacteria (1.52%); the dominant bacterial genera were *Prevotella* (8.08%), *Faecalibacterium* (7.05%), *unclassified_o__Eubacteriales* (4.86%), *Blautia* (4.54%), and *Phocaeicola* (4.47%); and the dominant bacterial species included *Faecalibacterium prausnitzii* (4.22%), *Prevotella* sp. (4.15%), *Clostridia bacterium* (3.71%), *Phocaeicola coprophilus* (2.80%), and *Blautia wexlerae* (2.73%) (Table S13). Similarly, employing LEfSe analysis to compare the relative abundance of fecal microbial taxa, no significant differences were observed at the phylum level among fecal bacteria. At the genus level, only two significantly different genera were identified between the groups—*Enterococcus* was enriched in the fecal microbiome of LADG calves, while *Anaerovorax* was enriched in the fecal microbiome of HADG calves (LDA > 2, *P* < 0.05; Fig. 6B, Table S14). Moreover, at the species level, six species, including *Parabacteroides* sp. AF48-14, *Bacterium 1XD8-76*, *Anaerovorax* sp. IOR16, *Parabacteroides distasonis*, *Candidatus* Fournierella merdipullorum, and *Candidatus* Parabacteroides faecavium, exhibited significantly higher relative abundances in the fecal microbiome of HADG calves (HADG-enriched fecal microbes) (LDA > 2, *P* < 0.05; Fig. 6C, Table S14). Conversely, only three species, including *Bifidobacterium pseudolongum*, *Enterococcus durans*, and *Faecalibacterium* sp. Marseille-Q0746, showed significant enrichment in the fecal microbiome of LADG calves (LADG-enriched fecal microbes) (LDA > 2, *P* < 0.05; Fig. 6C, Table S14). Subsequently, Spearman’s rank correlation analysis identified associations between different bacterial species and ADG. Notably, *B. pseudolongum*, *E. durans*, and *F.* sp. Marseille-Q0746 were found to be negatively associated with ADG (ADG-associated fecal microbes) (*R* <  − 0.50, *P* < 0.05; Fig. S11).

### Functional profiles of the fecal microbiome and differential functions between the HADG and LADG calves

The fecal microbiome was analyzed for functional characteristics using Kyoto Encyclopedia of Genes and Genomes (KEGG) profiles and carbohydrate-active enzymes (CAZymes) profiles. The PCA plots revealed no significant separation between the HADG and LADG calves based on KEGG level-3 pathways or family-level CAZymes (*P* > 0.05; Fig. S12). The distribution of KEGG level-1 pathways within the fecal microbiome of 16 calves, comprising “metabolism” (51.23%), “genetic information processing” (17.92%), “environmental information processing” (12.12%), “human diseases” (8.91%), “cellular processes” (7.36%), and “organismal systems” (2.47%), did not exhibit significant differences between the two groups (Fig. S13). However, at the KEGG level-2 pathways, “cell growth and death” and “immune disease” were notably enriched in the fecal microbiome of HADG calves compared to the LADG calves (LDA > 2, *P* < 0.05; Fig. 6D). Furthermore, only one KEGG level-3 pathway, “primary immunodeficiency” (ko05340), displayed significant enrichment in the fecal microbiome of HADG calves compared to the LADG calves (LDA > 2, *P* < 0.05; Fig. 6E). Notably, when evaluating KEGG modules, nine were enriched in the fecal microbiome of HADG calves, while one was enriched in the fecal microbiome of LADG calves (LDA > 2, *P* < 0.05; Fig. S14).

Regarding CAZyme profiles within the fecal microbiome of 16 calves, the distribution of class-level CAZymes encompassed GHs (50.97%), GTs (29.10%), CEs (10.98%), CBMs (5.98%), AAs (2.34%), PLs (0.52%), and CMs (0.12%), none of which exhibited significant differences between the two groups (Fig. S15A). However, at the family-level CAZymes, 14 significantly different CAZymes were identified. Of these, nine (7 GHs, 1 GT, and 1 CE) were enriched in the fecal microbiome of LADG calves, while five (4 GHs and 1 GT) were more abundant in the fecal microbiome of HADG calves (LDA > 2, *P* < 0.05; Fig. S15B).

### Fecal metabolome profiling differed between higher and lower performance calves

**Fig. 7:**
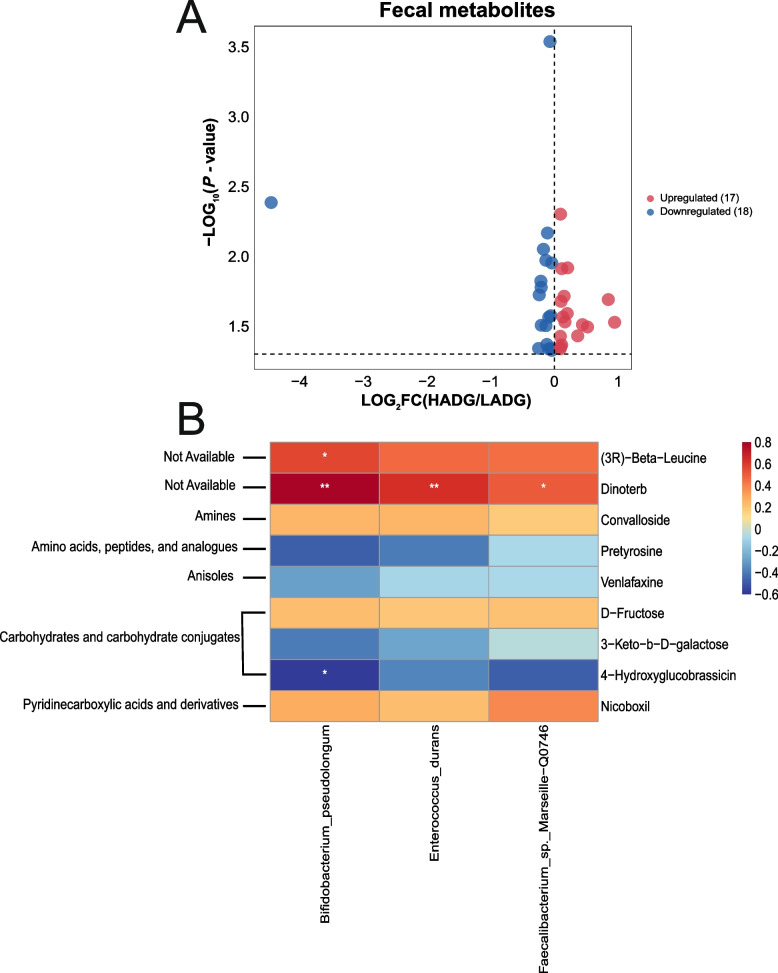
Fecal metabolites and their relationship with fecal microbes. **A** Volcano plot of fecal differential metabolites (VIP > 1, *P* < 0.05). **B** Correlations between the ADG-associated fecal metabolites and the ADG-associated fecal microbes (Spearman’s correlation). * Means Spearman’s |r| > 0.50 and *P* < 0.05, ** means Spearman’s |r| > 0.50 and *P* < 0.01. HADG, higher average daily gain group; LADG, lower average daily gain group

A total of 35 significantly different fecal metabolites were identified between the two groups, comprising 17 upregulated and 18 downregulated metabolites in the HADG calves (*P* < 0.05, VIP > 1; Fig. 7A, Table S15). Correlation analysis between these fecal metabolites and ADG revealed that nine metabolites were significantly associated with ADG (ADG-associated fecal metabolites) (*R* > |0.50|, *P* < 0.05; Table S16). Among these, four metabolites — pretyrosine, 3-keto-b-D-galactose, 4-hydroxyglucobrassicin, and venlafaxine — were notably higher in the HADG group and positively correlated with ADG (HADG-associated fecal metabolites) (Table S15; Table S16). Conversely, five metabolites, including convalloside, D-fructose, (3R)-beta-leucine, nicoboxil, and dinoterb, were elevated in the LADG group and negatively associated with ADG (LADG-associated fecal metabolites) (Table S15; Table S16).

Further analysis of the relationship between ADG-associated fecal microbes and ADG-associated fecal metabolites revealed that all LADG-enriched fecal microbes exhibited positive correlations with dinoterb (*R* > 0.50, *P* < 0.05; Fig. 7B). Additionally, *B. pseudolongum* showed a negative association with 4-hydroxyglucobrassicin (*R* <  − 0.50, *P* < 0.05; Fig. 7B).

### Plasma metabolome and relationships between rumen microbiome, fecal microbiome, and plasma metabolome

**Fig. 8:**
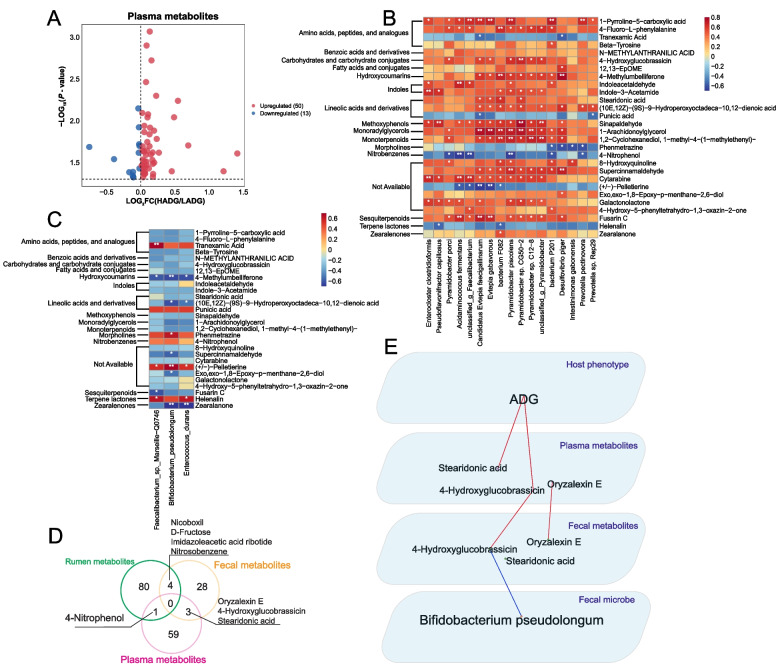
Plasma metabolites and their relationship with gastrointestinal microbes. **A** Volcano plot of plasma differential metabolites (VIP > 1, *P* < 0.05). **B** Correlations between the ADG-associated rumen microbes and the ADG-associated plasma metabolites. * Means Spearman’s |r| > 0.50 and *P* < 0.05, ** means Spearman’s |r| > 0.50 and *P* < 0.01. **C** Correlations between the ADG-associated fecal microbes and the ADG-associated plasma metabolites. * Means Spearman’s |r| > 0.50 and *P* < 0.05, ** means Spearman’s |r| > 0.50 and *P* < 0.01. **D** The Venn diagram shows significantly different metabolites in rumen, feces, and plasma. **E** Multiplex networks revealed the relationships between fecal microbes, fecal metabolites, plasma metabolites, and host phenotype. Lines between two nodes represent the correlation, with a red line indicating a positive correlation and a blue line indicating a negative correlation (Spearman’s |r| > 0.50 and *P* < 0.05). HADG, higher average daily gain group; LADG, lower average daily gain group

A total of 63 significantly different metabolites were identified in the plasma metabolome. Among them, 50 metabolites were enriched in the plasma of HADG calves, while 13 metabolites were enriched in the plasma of LADG calves (*P* < 0.05, VIP > 1; Fig. 8A, Table S17). Spearman’s rank correlation analysis, employing ADG and the differential plasma metabolites, revealed associations (ADG-associated plasma metabolites) (*R* > |0.50|, *P* < 0.05; Table S18). Specifically, 22 metabolites such as 4-hydroxyglucobrassicin, 1-pyrroline-5-carboxylic acid, stearidonic acid, and 4-fluoro-L-phenylalanine were notably higher in HADG and positively correlated with ADG (HADG-associated plasma metabolites). Conversely, six metabolites were significantly higher in LADG and negatively associated with ADG (LADG-associated plasma metabolites) (Table S17; Table S18).

To explore the relationships between gastrointestinal microbes and plasma metabolites, separate Spearman’s rank correlation analyses were conducted for ADG-associated rumen microbes and ADG-associated plasma metabolites, as well as for ADG-associated fecal microbes and ADG-associated plasma metabolites. The results revealed extensive connections between rumen microbes and plasma metabolites, notably with nearly all HADG-enriched rumen microbes exhibiting positive correlations with HADG-associated plasma metabolites (Fig. 8B). Particularly, the five *Pyramidobacter* species (*P. piscolens*, *P.* sp. CG50 − 2, *P.* sp. C12 − 8, *unclassified_g_Pyramidobacter*, and *P. porci*) showed positive correlations with 4-hydroxyglucobrassicin (*R* > 0.50, *P* < 0.05; Fig. 8B). Additionally, these five *Pyramidobacter* species and *A. fermentans* were positively associated with 4-fluoro-L-phenylalanine (*R* > 0.50, *P* < 0.05; Fig. 8B). Three rumen microbes (*P. piscolens*, *P. porci*, and *A. fermentans*) were also positively linked to 1-pyrroline-5-carboxylic acid (*R* > 0.50, *P* < 0.05; Fig. 8B). Furthermore, four rumen microbes (*P.* sp. CG50 − 2, C.* E. faecigallinarum*, *E. gabavorous*, and *bacterium F082*) exhibited positive relationships with stearidonic acid (*R* > 0.50, *P* < 0.05; Fig. 8B). Notably, *Desulfovibrio piger* was positively associated with 12,13-EpOME (*R* > 0.50, *P* < 0.05; Fig. 8B). In contrast, there appeared to be fewer associations between ADG-associated fecal microbes and ADG-associated plasma metabolites (Fig. 8C).

### Transfer of hindgut metabolites to plasma

Utilizing Venn diagrams, we observed shared differential metabolites between the rumen metabolome or fecal metabolome and the plasma metabolome (Fig. 8D), suggesting a potential transfer of these metabolites from the digestive tract into the plasma. Subsequently, multiplex networks were established to scrutinize the relationships among gastrointestinal microbes, gastrointestinal metabolites, plasma metabolites, and ADG, revealing the existence of one complete chain of regulation (Fig. 8E). Notably, a negative correlation was evident between the fecal microbe *B. pseudolongum* and 4-hydroxyglucobrassicin in feces (*R* <  − 0.50, *P* < 0.05; Fig. 8E). Furthermore, 4-hydroxyglucobrassicin in feces exhibited a positive correlation with 4-hydroxyglucobrassicin in plasma, while 4-hydroxyglucobrassicin in plasma showed a positive correlation with ADG (*R* > 0.50, *P* < 0.05; Fig. 8E).

### In vitro culture to obtain ADG-associated rumen microbes and in vitro fermentation experiment

By 16S rRNA sequencing and subsequent sequence comparison with the NCBI database using BLAST, the selected isolate was found to have the highest identity (98.94% identity) with *Acidaminococcus fermentans* strain 9-D20-63. We named the isolated strain as *A. fermentans* P41 (Table S19).

**Fig. 9:**
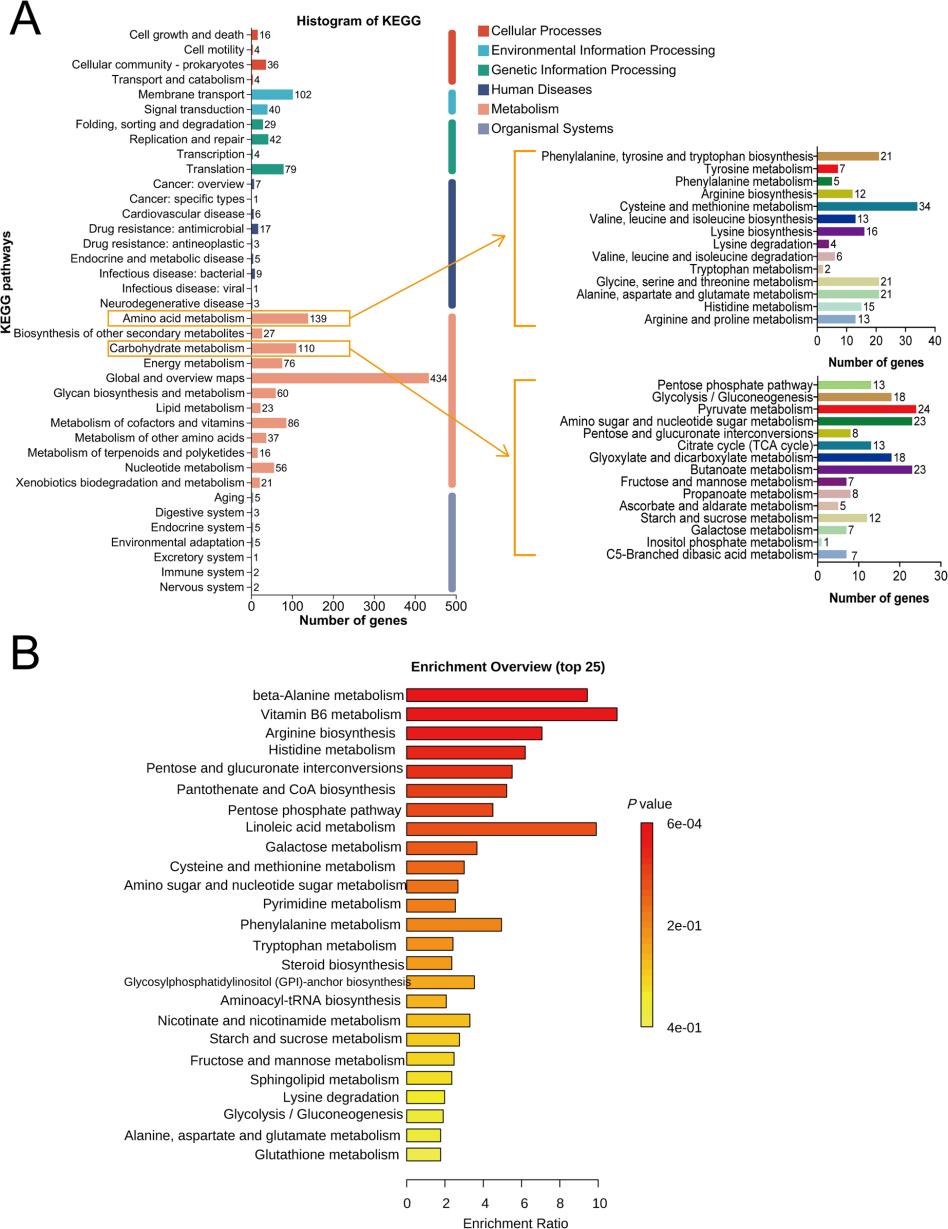
Genomic annotation results and metabolome of *Acidaminococcus fermentans* P41. **A** The number of genes of *Acidaminococcus fermentans* P41 in KEGG pathways. **B** Enrichment analysis based on differential metabolites from *Acidaminococcus fermentans* P41 fermentation

We determined that the *A. fermentans* P41 genome is 2,374,376 bp, with a GC content of 55.55%, a total of 2184 coding sequences, 59 tRNAs, 4 rRNAs, and 28 sRNAs (Fig. S16). The KEGG annotation indicated that most of the annotated genes were involved in global and overview maps (434 genes), amino acid metabolism (139 genes), and carbohydrate metabolism (110 genes). Further analysis of carbohydrate metabolism and amino acid metabolism showed that the annotated genes were also involved in some key KEGG level 3 pathways such as glycolysis/gluconeogenesis (18 genes), pyruvate metabolism (24 genes), butanoate metabolism (23 genes), fructose and mannose metabolism (7 genes), starch and sucrose metabolism (12 genes), cysteine and methionine metabolism (34 genes), and phenylalanine, tyrosine, and tryptophan biosynthesis (21genes) (Fig. 9A).

The metabolic profile of *A. fermentans* P41 unveiled 89 differential metabolites, with 48 metabolites upregulated and 41 downregulated in the AFP cultures (VIP > 1, *P* < 0.05; Table S20). Specifically, this strain exhibits a diverse range of activities, synthesizing various amino acids, peptides, and analogs like N-acetyl-L-phenylalanine, L-aspartic acid, pyroglutamic acid, pipecolic acid, beta-leucinebeta-alanyl-L-arginine, and L-alanyl-gamma-D-glutamyl-L-lysine (FC > 1, VIP > 1, *P* < 0.05; Table S20). Conversely, it utilizes compounds like L-methionine, homo-L-arginine, and D-tryptophan (FC < 1, VIP > 1, *P* < 0.05; Table S20). Additionally, *A. fermentans* P41 displays the ability to metabolize carbohydrates and carbohydrate conjugates, including L-fucose, D-glucose 1-phosphate, and D-ribose (FC < 1, VIP > 1, *P* < 0.05; Table S20).

Enrichment analysis further elucidated the involvement of *A. fermentans P41* in carbohydrate and amino acid metabolism pathways. Notably, pathways like beta-alanine metabolism, arginine biosynthesis, histidine metabolism, phenylalanine metabolism, tryptophan metabolism, lysine degradation, alanine, aspartate, and glutamate metabolism, starch and sucrose metabolism, glycolysis/gluconeogenesis, and fructose and mannose metabolism were significantly enriched (*P* < 0.05; Fig. 9B). Collectively, these findings confirm the active engagement of *A. fermentans P41* in carbohydrate and amino acid metabolism, substantially corroborating our observations in the rumen.

Subsequent acid production tests unequivocally confirmed its capability to generate acetate and butyrate, affirming the observed rumen microbiome characteristics and whole genome sequencing (Fig. S17). Furthermore, the in vitro fermentation experiment indicated that the addition of *A. fermentans P41* increased the proportion of butyrate in the rumen fluid, irrespective of the substrate used (*P* < 0.05; Fig. S18).

## Discussion

In the present study, the 16 preweaning calves under the same management and dietary conditions were used to elucidate the relationship between the gastrointestinal microbes and host growth rate and revealed that the gastrointestinal microbes of preweaning calves affected host ADG. The in vitro cultivation of ADG-associated rumen microbes and in vitro fermentation experiment further validate our discoveries and provide strain resources for modulating calf gastrointestinal microbes to improve ADG.

Rumen bacteria comprise the predominant microbial domain in the calves’ rumen [38], playing a pivotal role in driving rumen fermentation [39]. Our study unveiled variations in the relative abundance of rumen bacteria at distinct taxonomic levels between HADG and LADG calves. Particularly notable were the differences observed at the bacterial genus and species levels, with almost all differential taxa enriched in the rumen microbiome of HADG calves, and the majority of these differential taxa belong to less abundant taxa. Specifically, at the species level, numerous species that exhibited significantly higher relative abundances in the rumen microbiome of HADG calves belonged to the *Pyramidobacter* genus. *Pyramidobacter*, recognized for its acid-producing strains, generates diverse end products including acetate, isovalerate, propionate, butyrate, succinate, and phenylacetic acid [40, 41]. This genus is also associated with rumen vitamin B1 synthesis and rumen development [42]. The *Pyramidobacte*r species, such as *P.* sp. C12-8, *P.* sp. CG50-2, *P. porci*, *unclassified_g_Pyramidobacter*, and *P. piscolens*, were enriched in the rumen of HADG calves and act as butyrate-producing bacteria in the rumen. They showed positive relationships with proportion of butyrate and ADG, suggesting that they may be involved in VFA biosynthesis and growth promotion. Furthermore, *Prevotella* genus was found to be associated with butyrate production and growth performance in ruminants [43, 44]. Our findings, showing that the relative abundances of *P.* sp. Rep29 and *Prevotella pectinovora* were positively related to ADG and the proportion of butyrate, provided additional support for this association. A particular focus should be placed on *A. fermentans*, identified as an HADG-enriched rumen microbe, which showed correlations with ADG and the proportion of butyrate. Published studies suggest that *A. fermentans* not only produces acetate and butyrate but also has the capability to eliminate harmful metabolites in rumen fermentation [45, 46]. In our efforts to obtain rumen microbes capable of influencing host ADG, we conducted extensive in vitro cultivation. However, due to limitations in cultivation techniques and our understanding of rumen microbes, we obtained only a strain of *A. fermentans* P41. Whole genome sequencing revealed that it has genes involved in butanoate metabolism, and acid production tests confirmed its ability to produce acetate and butyrate, further affirming our findings and enhancing result credibility. Future research should concentrate on the large-scale isolation and cultivation of ADG-associated rumen microbes. This approach not only validates our findings but also opens avenues for manipulating rumen microbes in calf studies.

The functional stability of the rumen microbiome is widely acknowledged to surpass its taxonomic composition [47]. Notably, the majority of KEGG functions displayed higher activity in the rumen of HADG calves, consistent with the observed taxonomic composition. Specifically, KEGG functions linked to carbohydrate degradation and volatile fatty acid formation were significantly enriched in the rumen microbiome of HADG calves. These included pathways such as “fructose and mannose metabolism,” “starch and sucrose metabolism,” and “glycolysis,” suggesting that the rumen microbiome of HADG calves exhibits an enhanced capacity for carbohydrate degradation, leading to increased hydrolytic products and pyruvate production. Interestingly, a substantial reduction in the rumen metabolite D-fructose in HADG calves compared to LADG calves directly corroborates the enhanced carbohydrate degradation in the rumen microbiome of HADG calves. Carbohydrate-degrading enzymes (GHs, PLs, and CEs) play a role in this process [48]. The enrichment of CAZymes, including 4 CEs, 55 GHs, and 2 PLs in the rumen microbiome of HADG calves, provides additional evidence of their heightened capability for carbohydrate degradation. Regarding volatile fatty acid (VFA) formation, the enrichment of the “butanoate metabolism” pathway in the rumen microbiome of HADG calves suggests a preference for butyrate production. This finding aligns with the direct test for rumen VFAs, providing concrete evidence of a higher proportion of butyrate in the rumen of HADG calves. Butyrate is known to promote gastrointestinal tract development, including the forestomach and lower gastrointestinal tract (abdominal cavity, intestines, and pancreas) [49, 50]. Studies have shown that butyrate supplementation to preweaning calves increases their ADG [51], implying that the higher proportion of butyrate in the rumen of HADG calves may contribute to their elevated ADG. Multiplex networks unveiled that ADG-associated rumen microbes (such as *P.* sp. C12-8, P*.* sp. CG50-2, *P. porci*, *unclassified_g_Pyramidobacter*, *P. piscolens*, *A. fermentans*, *P.* sp. Rep29, and *P. pectinovora*) in HADG calves led to the enrichment of six KEGG pathways associated with carbohydrate degradation and butyrate production. This, in turn, resulted in an increased proportion of butyrate, potentially contributing to the observed higher ADG. The results of the whole genome sequencing and metabolome of *A. fermentans* P41 support these findings, revealing its involvement in carbohydrate metabolism pathways (“starch and sucrose metabolism,” “glycolysis,” and “fructose and mannose metabolism”) and its ability to produce butyrate. The more direct evidence was that in vitro fermentation experiments showed that the addition of *A. fermentans* P41 increased the proportion of butyrate in the rumen fluid.

Studies have underscored the substantial role of amino acid metabolism in determining individualized dairy cow performance [11]. Within the “global and overview maps” pathway, heightened activity in the “biosynthesis of amino acids” pathway was evident in the rumen of HADG calves. Specifically, seven pathways associated with amino acid synthesis, including “glycine, serine, and threonine metabolism,” “phenylalanine, tyrosine, and tryptophan biosynthesis,” “valine, leucine, and isoleucine biosynthesis,” “lysine biosynthesis,” “histidine metabolism,” “alanine, aspartate, and glutamate metabolism,” and “arginine biosynthesis,” were enriched in the rumen microbiome of HADG calves. Protein degradation leads to the breakdown of true protein into amino acids and ammonia, utilized by ruminal microbes for microbial protein (MCP) synthesis [52]. The enrichment of these amino acid biosynthetic pathways in the rumen of HADG calves suggests a potential increase in microbial protein synthesis, ultimately digested in the small intestine [53]. Notably, amino acids like methionine, lysine, isoleucine, threonine, and histidine might be limiting for young calves [53, 54]. The enrichment of these pathways implies that the synthesized microbial protein in the rumen of HADG calves may better meet the calves’ amino acid composition requirements. Multiplex networks also unveiled that rumen microbes regulate the host phenotype through amino acid metabolism. All ADG-associated rumen microbes led to more active amino acid synthesis pathways in the rumen of HADG calves, potentially better meeting the host’s nutritional requirements and ultimately enhancing ADG. The whole genome sequencing and metabolome of *A. fermentans* P41 revealed its involvement in pathways like “arginine biosynthesis,” “histidine metabolism,” and “alanine, aspartate, and glutamate metabolism,” aligning with findings from the rumen microbiome. Moreover, *A. fermentans* P41 can generate various amino acids, peptides, and analogs like L-aspartic acid, beta-leucine, N-acetyl-L-phenylalanine, beta-alanyl-L-arginine, and L-alanyl-gamma-D-glutamyl-L-lysine. These compounds might be utilized by the host or other ruminal microbes, potentially fostering the growth and development of calves. An additional enrichment observed in the rumen of HADG calves was in “vitamin B6 metabolism.” B vitamins serve as essential coenzymes for enzymes involved in carbohydrate, lipid, protein, and nucleic acid metabolism [55]. Thus, the enrichment of “vitamin B6 metabolism” suggests a potentially more active metabolism in the rumen of HADG calves, further supported by our preceding discussions.

The current study systematically delved into the connections between rumen microbes and rumen metabolites. In ruminants, the rumen stands as a pivotal digestive organ where microbes break down ingested feed, supplying nutrients to the host [56]. Our findings underscored this crucial role. The numerous negative correlations observed between rumen microbes and rumen metabolites suggest an active involvement of rumen microbes in metabolite degradation within the rumen. Furthermore, the associations between rumen metabolites and rumen microbes effectively mirror the functional variations within the rumen microbiome. Several rumen microbes associated with ADG, including *P. piscolens*, *P.* sp. C12-8, and *unclassified_g_Pyramidobacter*, displayed negative correlations with carbohydrates and carbohydrate conjugates (D-fructose), indicating their potential ability in carbohydrate degradation. A similar trend emerged in fatty acids and conjugates, where *P. piscolens*, *bacterium P201*, and *bacterium F082* (ADG-associated rumen microbes) showed negative associations with 8,9-epoxyeicosatrienoic acid. In contrast, *bacterium P201* and *bacterium F082* (ADG-associated rumen microbes) exhibited positive correlations with amino acids, peptides, and analogs (frangulanine). These correlations underscore the advantageous capability of the HADG rumen microbiome in amino acid synthesis. Overall, our data highlight the significance of certain ADG-associated rumen microbes, such as *P. piscolens* and *bacterium P201*, in nutrient digestion, beneficial metabolite production, and their potential contribution to calf growth.

In contrast to the significant differences observed in the rumen microbiome, variations within the fecal microbiome between the two groups were relatively minor. No discernible differences were identified at the domain level. Given the prevalence of bacteria in the fecal microbiome, our analysis primarily focused on these microbial taxa. At the species level, most of the distinct bacteria were enriched in the fecal microbiome of HADG calves. Specifically, *P. distasonis*, a representative strain of the *Parabacteroides* genus commonly found in various species’ gastrointestinal tracts [57], plays a crucial role as a core member of gut microbes, offering numerous beneficial properties to its host [58, 59]. Considering the association of the *Parabacteroides* genus with host health [57], the enrichment of *P.* sp. AF48-14 in the hindgut potentially contributes favorably to the growth and development of HADG calves. The combined enrichment of the two *Parabacteroides* species in the hindgut may positively influence the growth and development of HADG calves. The *Enterococcus* genus is known for its beneficial impact on animal health [60], indicating that *E. durans* might contribute positively to the health of LADG calves. *B. pseudolongum* has the potential to alter the host gut microbiota and offer advantages in the host’s digestive processes [61]. However, in this study, *E. durans* and *B. pseudolongum* were found to be negatively correlated with the ADG in calves. In the subsequent analysis of the metabolome, we identified a potential mechanism through which *B. pseudolongum* may influence ADG. Surprisingly, only one KEGG level-3 pathway displayed significant disparity between the two groups in the functional aspect of the fecal microbiome. The fermentation capacity of the hindgut represents approximately 14% of that in the rumen [62], underscoring the greater impact of rumen fermentation on the host compared to hindgut fermentation. This implies that differences in host growth performance are more likely to be attributed to rumen fermentation driven by rumen microbes rather than hindgut fermentation driven by hindgut microbes. This is also reflected in our results. In our study, the PCA plots revealed separation in both the composition of rumen bacteria and the distribution of rumen KEGG pathways between the two groups. Conversely, the differences in microbial composition and function within the hindgut microbiome between the two groups were relatively minor. The lower correlations between fecal microbes and fecal metabolites also suggested potentially lower metabolic activity in the hindgut.

The host metabolome not only contributes to the host phenotype but also shows potential interactions with both the rumen microbiome and hindgut microbiome [63, 64]. Overall, widespread positive correlations were observed between rumen microbes and plasma metabolites. Most crucial metabolites, such as amino acids, peptides, analogs, linoleic acids, and derivatives, as well as carbohydrates and carbohydrate conjugates, were notably enriched in the plasma of HADG calves. Specifically, 1-pyrroline-5-carboxylic acid, an intermediate involved in the interconversions among proline, ornithine, and glutamate, stimulates glucose oxidation through the hexose monophosphate-pentose pathway [65]. Positive correlations were identified between rumen microbes (such as *P. piscolens*, *P. porci*, and *A. fermentans*) and 1-pyrroline-5-carboxylic acid (HADG-associated plasma metabolites). This suggests that these three rumen microbes may be involved in regulating 1-pyrroline-5-carboxylic acid, potentially contributing to its enrichment in plasma. Elevated levels of 1-pyrroline-5-carboxylic acid may promote enhanced energy utilization, ultimately enhancing the growth rate of HADG calves. A similar scenario was observed with 4-fluoro-L-phenylalanine (HADG-associated plasma metabolites). The three aforementioned rumen microbes also showed positive correlations with 4-fluoro-L-phenylalanine that can be converted to tyrosine by liver enzymes [66]. This suggests that these rumen microbes potentially affected the accumulation of 4-fluoro-L-phenylalanine into plasma, which may have affected the ADG. Regarding linoleic acids and derivatives, such as stearidonic acid (HADG-associated plasma metabolites), which derive from linolenic acid bioconversion, these compounds have potential health effects on the host [67]. The discovery of four rumen microbes (*P.* sp. CG50 − 2, *Ca.* E. faecigallinarum, *E. gabavorous*, and *bacterium F082*) positively correlating with stearidonic acid suggests their potential role in regulating stearidonic acid in plasma, promoting host health, and enhancing the host's growth rate. Additionally, concerning 4-hydroxyglucobrassicin (HADG-associated plasma metabolites), a glucosinolate, this compound is known for its role in plant defense and human health benefits [68]. Rumen microbes seem to influence the levels of 4-hydroxyglucobrassicin in plasma. The 4-hydroxyglucobrassicin is thought to be a compound of plant origin [69], so one question that arises is how it gets into the plasma. Interestingly, we also found 4-hydroxyglucobrassicin in fecal differential metabolites. Multiplex networks indicated a negative relationship between fecal *B. pseudolongum* and 4-hydroxyglucobrassicin in the hindgut of HADG calves. Studies have suggested that hindgut microbes tend to preferentially use carbohydrates [70–72]. This might imply that the lower relative abundance of *B. pseudolongum* in the hindgut consumed less 4-hydroxyglucobrassicin, leading to its accumulation in the hindgut and subsequent transfer to the plasma, exerting beneficial effects on the growth rate of HADG calves. Collectively, our data reveal that several ADG-associated rumen microbes, including *P.* sp. CG50 − 2, *P. piscolens*, *P. porci*, and *A. fermentans*, may play pivotal roles in regulating plasma metabolites positively associated with ADG. Regarding fecal microbes, *B. pseudolongum* may regulate the transfer of 4-hydroxyglucobrassicin from the hindgut to the plasma, thereby promoting calf growth.

It is crucial to emphasize that the variation in ADG among preweaning calves stems from the collective influence of various gastrointestinal microbes working in unison. While the acquisition of *A. fermentans* P41 through in vitro methods may represent one pivotal candidate influencing the host phenotype, several *Pyramidobacter* species within this study might have also played a significant role in regulating the ADG of preweaning calves. Despite extensive efforts in isolation and cultivation, unfortunately, we were unable to obtain any *Pyramidobacter* species. Future research endeavors should persist in large-scale microbial culture efforts and conduct additional feeding trials to validate these findings.

## Conclusion

To summarize, rumen microbes significantly impact both rumen fermentation and host metabolism, thereby influencing the average daily gain (ADG) of calves. Meanwhile, hindgut microbes affect calf growth by influencing the transfer of plant-derived metabolites from the hindgut to the plasma. Overall, the rumen microbiome showed differences in composition and function between preweaning calves with different ADG. Although the majority of the differential rumen bacterial species belong to less abundant taxa, they play an important role in regulating host phenotypes. Conversely, the fecal microbiome composition and function exhibited minimal to negligible variations across different growth rates in preweaning calves. Several specific rumen bacteria, including *P.* sp. C12-8, *P.* sp. CG50-2, *P. porci*, *unclassified_g_Pyramidobacter*, *P. piscolens*, and *A. fermentans*, were identified for their significant associations with calf growth. These variations in rumen microbes contributed to the functionalities of ruminal carbohydrate and amino acid metabolism, proportion of butyrate, and the presence of beneficial plasma metabolites in calves with higher ADG, potentially providing enhanced energy and nutrients for their growth. Our analysis of *A. fermentans* P41 obtained from in vitro cultures further supports these observations. Additionally, the lower relative abundance of *B. pseudolongum* in the hindgut of HADG calves may result in reduced consumption of the plant-derived metabolite 4-hydroxyglucobrassicin. This may lead to more 4-hydroxyglucobrassicin passing from the hindgut into the plasma, potentially enhancing the ADG in these calves. Therefore, our findings deepen the understanding of the influence of gastrointestinal microbes on the ADG of preweaning calves. They emphasize that rumen and fecal microbes have distinct roles in shaping the host phenotype and suggest that the continued cultivation of gastrointestinal microbes and further feeding trials are essential to validate their functions in future studies.

### Supplementary Information

Additional file 1: Table S1. Nutrient content of Starter and milk replacer (DM basis, %). Table S2. Composition of PYG medium. Table S3. Comparison of physiological parameters and rumen fermentation characteristics between LADG and HADG calves. Table S4. Summary of sequence data generated from rumen samples of 8 LADG and 8 HADG calves. Table S5. Wilcoxon rank sum test was used to compare the rumen microbial domain. Table S6. The relative abundances of the top 10 rumen bacterial phyla, genera and species between HADG and LADG calves. Table S7. The significant differences in rumen bacterial phyla, genera, and species between HADG and LADG calves. Table S8. The relative abundances of ruminal KEGG pathways between HADG and LADG calves. Table S9. Significantly different rumen metabolites between HADG and LADG calves. Table S10. ADG-associated metabolites in rumen. Table S11. Summary of sequence data generated from fecal samples of 8 LADG and 8 HADG calves. Table S12. Wilcoxon rank sum test was used to compare the fecal microbial domain. Table S13. The relative abundances of the top 10 feces bacterial phyla, genera and species between HADG and LADG calves. Table S14. The Significant differences in fecal bacterial genera and species between HADG and LADG calves. Table S15. Significantly different fecal metabolites between HADG and LADG calves. Table S16. ADG-associated metabolites in feces. Table S17. Significantly different plasma metabolites between HADG and LADG calves. Table S18. ADG-associated metabolites in plasme. Table S20**.** Differential metabolites due to by *Acidaminococcus fermentans P41* fermentation.

Additional file 2: Figure S1. Alpha and beta diversity of rumen microbes between HADG and LADG calves. A) Alpha diversity (Chao and Shannon indices) of rumen microbes between HADG and LADG calves. B) The Principal Component Analysis (PCA) based on rumen bacterial species level between HADG and LADG calves. 

Additional file 3: Figure S2. Heatmap displays the Spearman’s correlation coefficients among rumen microbes and host phenotypes. * means *R* > |0.50|, *P* < 0.05, ** means *R* > |0.50|, *P* < 0.01.

Additional file 4: Figure S3. The Principal Component Analysis (PCA) profile of rumen microbial function between HADG and LADG calves. A) The Principal Component Analysis (PCA) profile of ruminal KEGG level-3 pathways between HADG and LADG calves. B) The Principal Component Analysis (PCA) profile of ruminal family-level CAZymes between HADG and LADG calves.

Additional file 5: Figure S4. All significantly different KEGG pathways in the rumen. A) All significantly different KEGG level-2 pathways in the rumen (LDA > 2, *P* < 0.05). B) All significantly different KEGG level-3 pathways in the rumen (LDA > 2, *P *< 0.05).

Additional file 6: Figure S5. All significantly different KEGG modules in the rumen (LDA > 2, *P* < 0.05).

Additional file 7:  Figure S6. All significantly different KEGG enzymes in the rumen (LDA > 2, *P* < 0.05).

Additional file 8: Figure S7. All significantly different CAZymes in the rumen. A) The class-level CAZymes profiles in the rumen. * means LDA > 2, *P *< 0.05. B) All significantly different family-level CAZymes in the rumen (LDA > 2, *P *< 0.05).

Additional file 9: Figure S8. Multiplex networks revealed how rumen microbes influence host phenotypes by regulating carbohydrate metabolism. Lines between two nodes represent the correlation, with a red line indicating a positive correlation and a blue line indicating a negative correlation (Spearman’s |r| > 0.50 and *P* < 0.05).

Additional file 10: Figure S9. Multiplex networks revealed how rumen microbes influence host phenotypes by regulating amino acid metabolism. Lines between two nodes represent the correlation, with a red line indicating a positive correlation and a blue line indicating a negative correlation (Spearman’s |r| > 0.50 and *P* < 0.05).

Additional file 11: Figure S10. Alpha and beta diversity of fecal microbes between HADG and LADG calves. A) Alpha diversity (Chao and Shannon indices) of fecal microbes between HADG and LADG calves. B) The Principal Component Analysis (PCA) based on fecal bacterial species level between HADG and LADG calves.

Additional file 12: Figure S11. Heatmap displays the Spearman’s correlation coefficients among fecal microbes and host phenotypes. * means *R* > |0.50|, *P* < 0.05, ** means *R* > |0.50|, *P* < 0.01.

Additional file 13: Figure S12. The Principal Component Analysis (PCA) profile of fecal microbial function between HADG and LADG calves. A) The Principal Component Analysis (PCA) profile of fecal KEGG level-3 pathways between HADG and LADG calves. B) The Principal Component Analysis (PCA) profile of fecal family-level CAZymes between HADG and LADG calves.

Additional file 14: Figure S13. The distribution of KEGG pathways in the fecal microbiome. * means LDA > 2, *P* < 0.05.

Additional file 15: Figure S14. All significantly different KEGG modules in the feces (LDA > 2, *P* < 0.05).

Additional file 16: Figure S15. All significantly different CAZymes in the feces. A) The class-level CAZymes profiles in the feces. * means LDA > 2, *P *< 0.05. B) All significantly different family-level CAZymes in the feces (LDA > 2, *P *< 0.05).

Additional file 17: Table S19. 16s rRNA sequence of *Acidaminococcus fermentans P41*.

Additional file 18: Figure S16. Genomic architecture of *Acidaminococcus fermentans P41*. Each circle, from centre to the outside, represents the following features. The first circle represents the scale mark, the second circle represents GC skew, the third circle represents GC content, the fourth and seventh circles represent every COG to which each coding sequence (CDS) belongs, and the fifth and sixth circles represent the locations of CDS, tRNA and rRNA in the genome.

Additional file 19: Figure S17. Acid production capacity of *Acidaminococcus fermentans P41*. CON means PYG liquid medium; AFP means PYG liquid medium + *Acidaminococcus fermentans P41*.

Additional file 20: Figure S18. In vitro fermentation parameters. A) The in vitro fermentation parameters between the CON group and AFP group when using milk replacer as the substrate. B) The in vitro fermentation parameters between the CON group and AFP group when using milk replacer and starter as the substrate. C) The in vitro fermentation parameters between the CON group and AFP group when using starter as the substrate. CON means PYG liquid medium; AFP means PYG liquid medium + *Acidaminococcus fermentans P41*.

## Data Availability

The metagenome sequences were deposited into NCBI Sequence Read Archive (SRA) under the accession number of PRJNA1070330. For whole genome sequencing raw data, the submission number is SUB14124175, the bioproject accession is PRJNA1062104, and the biosample accession is SAMN39288640.
