# Supplementary figures and images for: Rumen and hindgut microbiome regulate average daily gain of preweaning Holstein heifer calves in different ways
Source: Microbiome. 2024 Jul 19;12:131. doi: 10.1186/s40168-024-01844-7 (PMC11264748; doi:10.1186/s40168-024-01844-7)

A

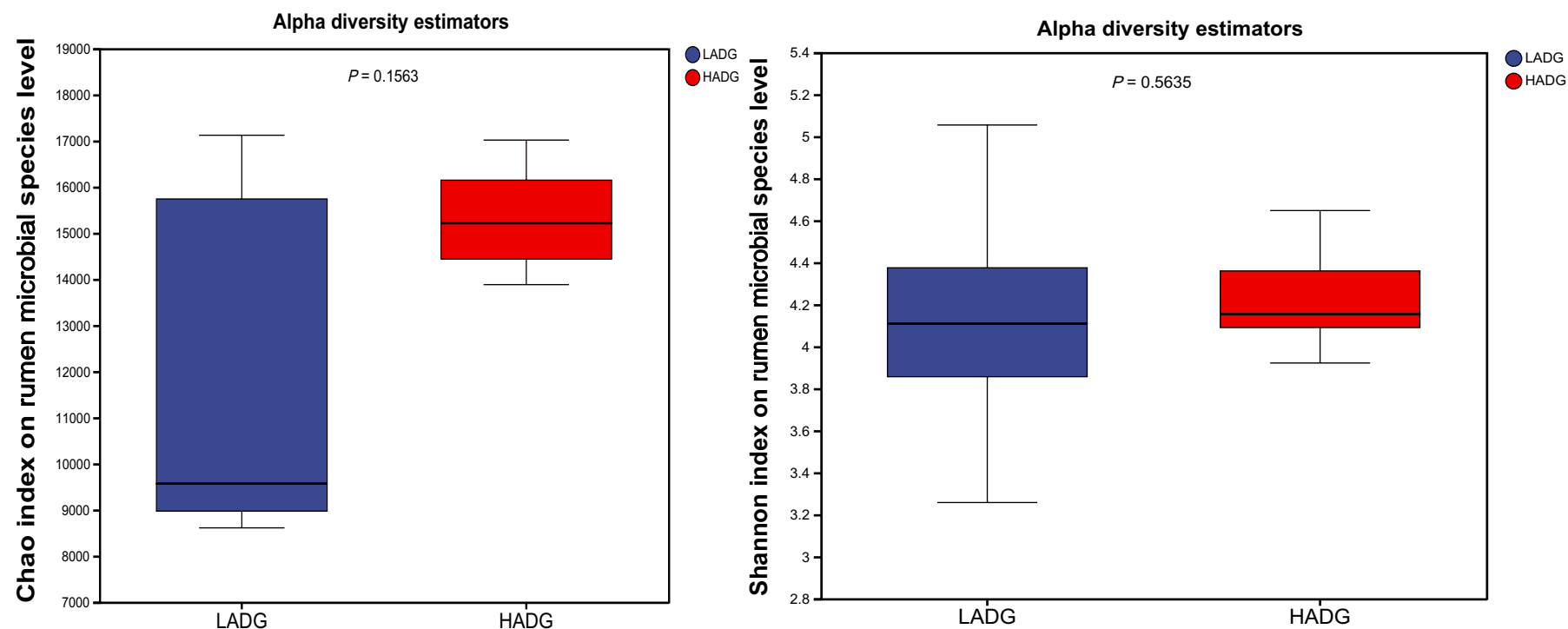

B

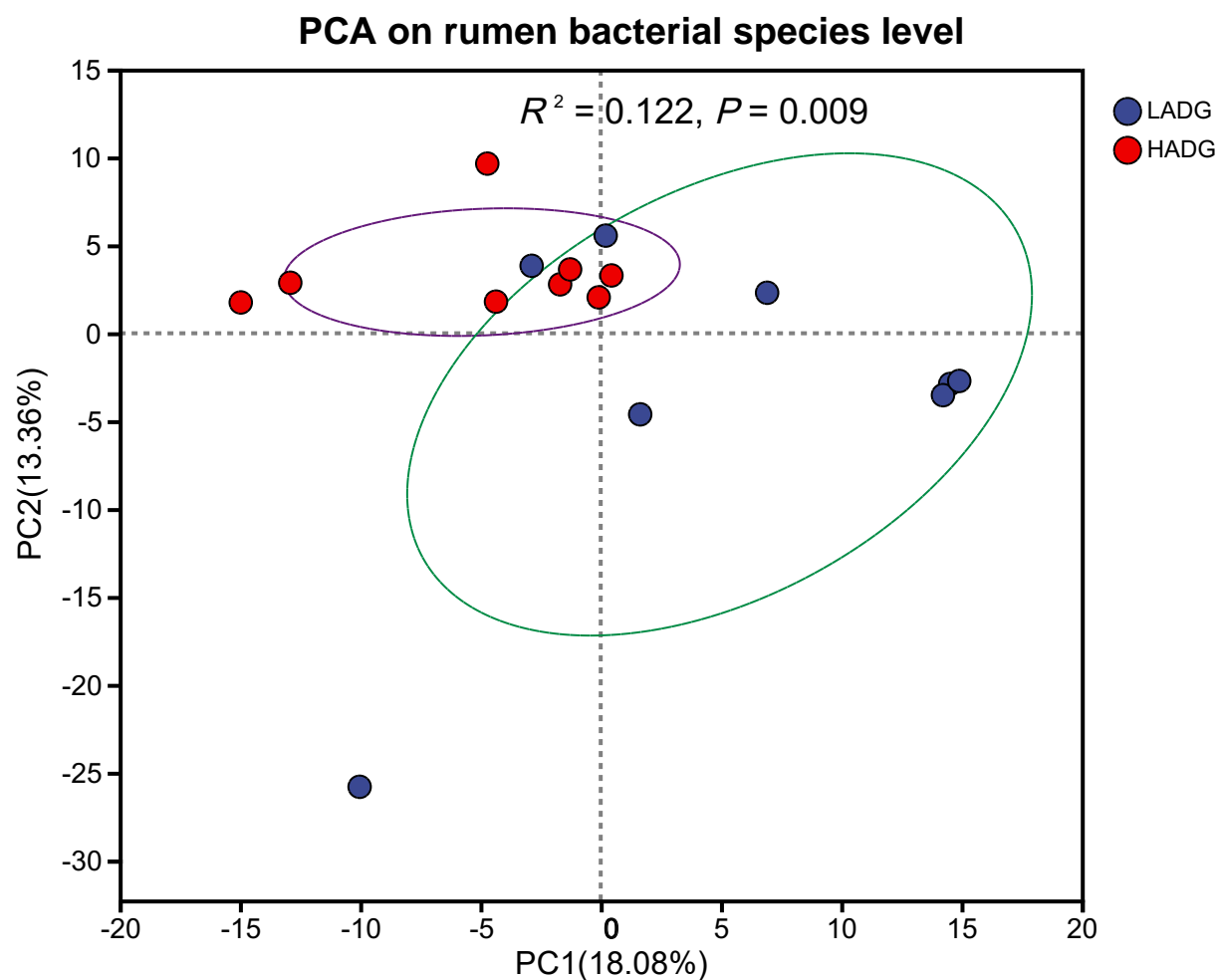

Supplement: Supplementary file 3 — Additional file 2: Figure S1. Alpha and beta diversity of rumen microbes between HADG and LADG calves. A) Alpha diversity (Chao and Shannon indices) of rumen microbes between HADG and LADG calves. B) The Principal Component Analysis (PCA) based on rumen bacterial species level between HADG and LADG calves. [file 40168_2024_1844_MOESM2_ESM.pdf]

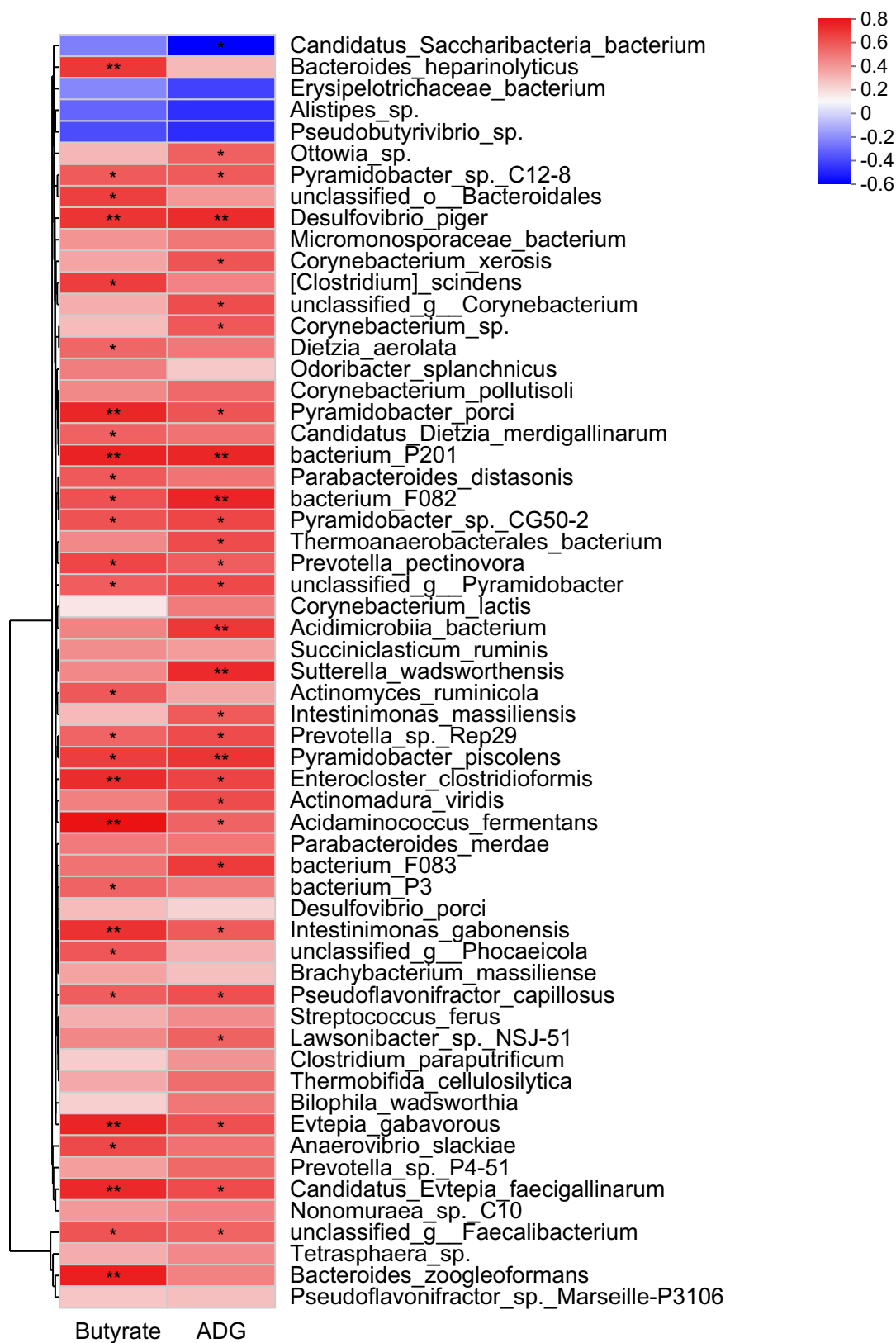

Supplement: Supplementary file 4 — Additional file 3: Figure S2. Heatmap displays the Spearman’s correlation coefficients among rumen microbes and host phenotypes. * means R > |0.50|, P < 0.05, ** means R > |0.50|, P < 0.01. [file 40168_2024_1844_MOESM3_ESM.pdf]

**A**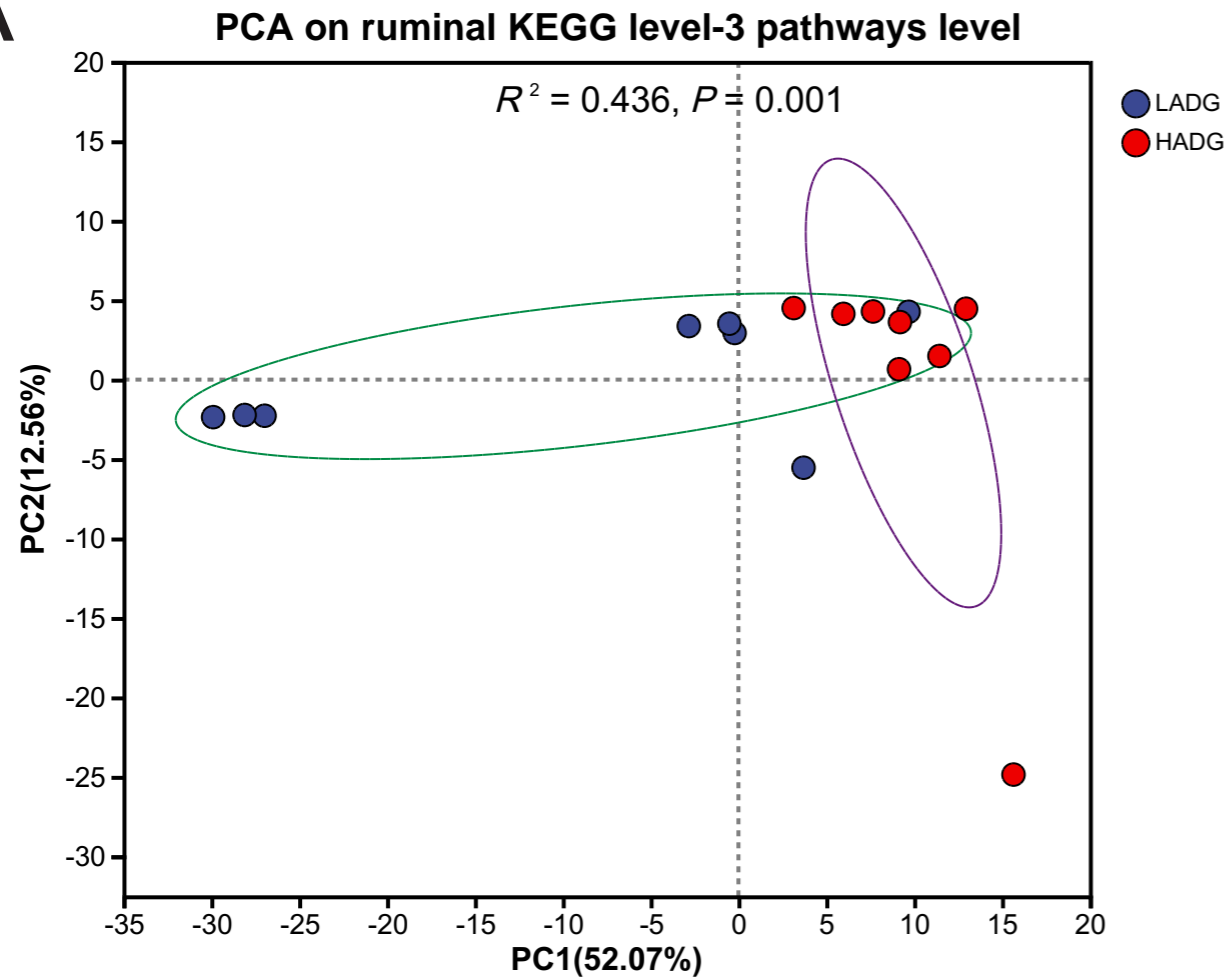**B**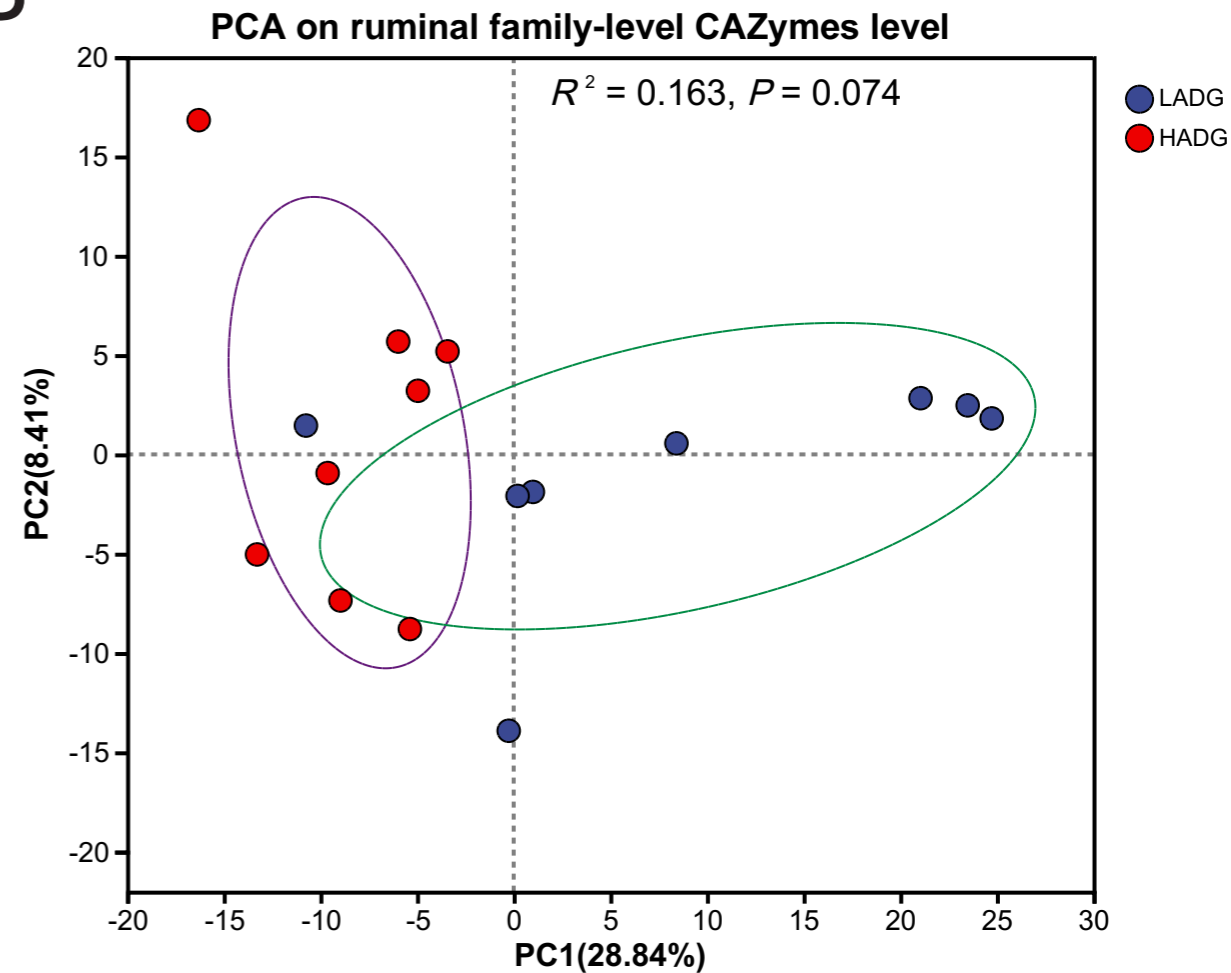

Supplement: Supplementary file 5 — Additional file 4: Figure S3. The Principal Component Analysis (PCA) profile of rumen microbial function between HADG and LADG calves. A) The Principal Component Analysis (PCA) profile of ruminal KEGG level-3 pathways between HADG and LADG calves. B) The Principal Component Analysis (PCA) profile of ruminal family-level CAZymes between HADG and LADG calves. [file 40168_2024_1844_MOESM4_ESM.pdf]

A

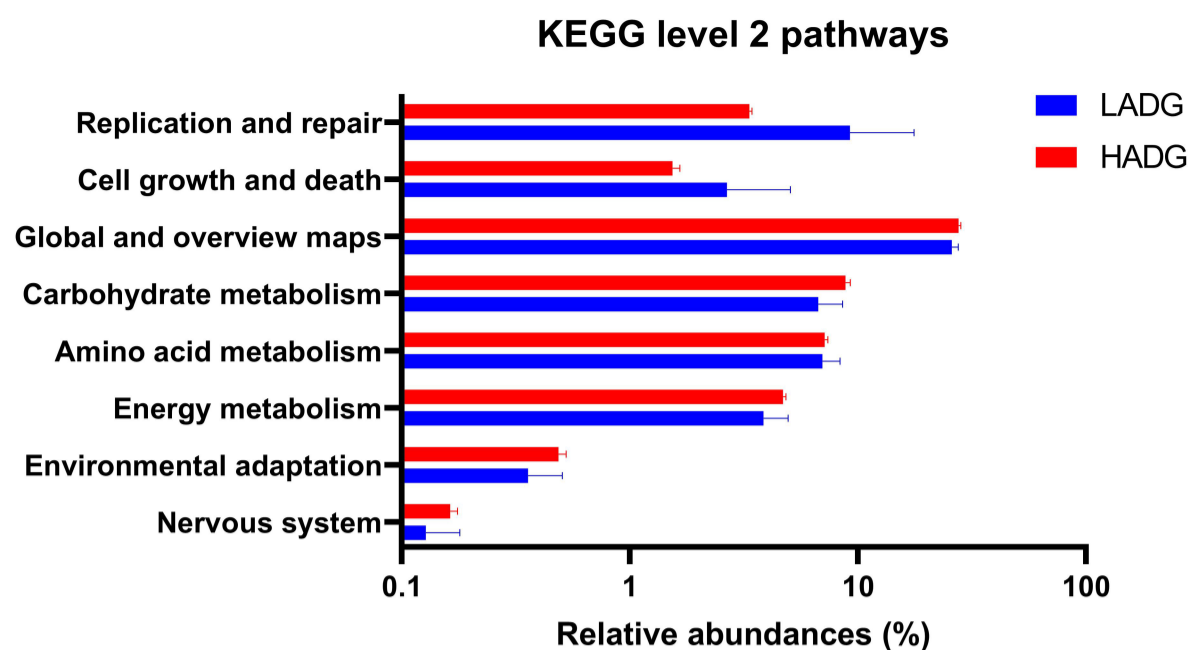

B

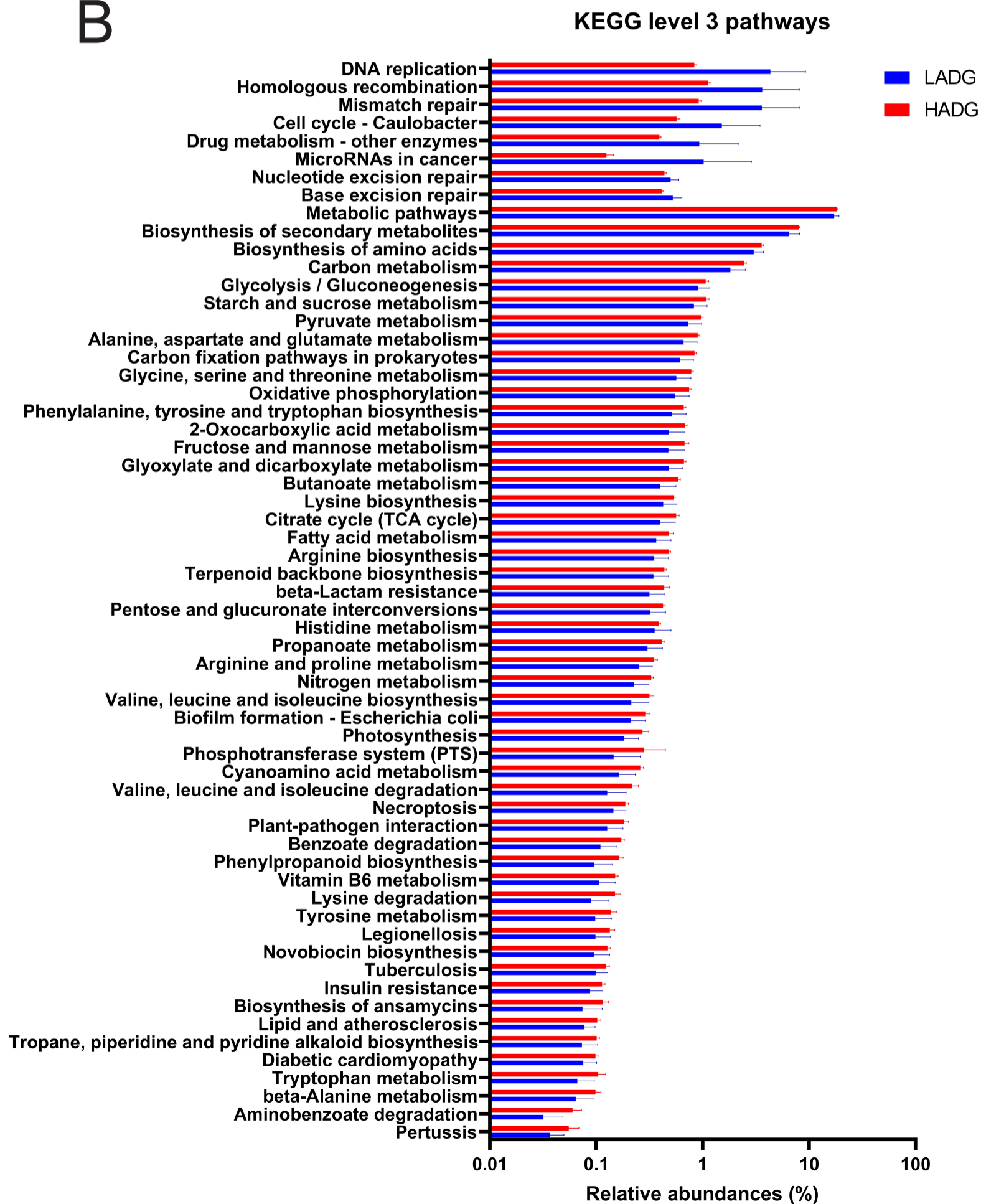

Supplement: Supplementary file 6 — Additional file 5: Figure S4. All significantly different KEGG pathways in the rumen. A) All significantly different KEGG level-2 pathways in the rumen (LDA > 2, P < 0.05). B) All significantly different KEGG level-3 pathways in the rumen (LDA > 2, P < 0.05). [file 40168_2024_1844_MOESM5_ESM.pdf]

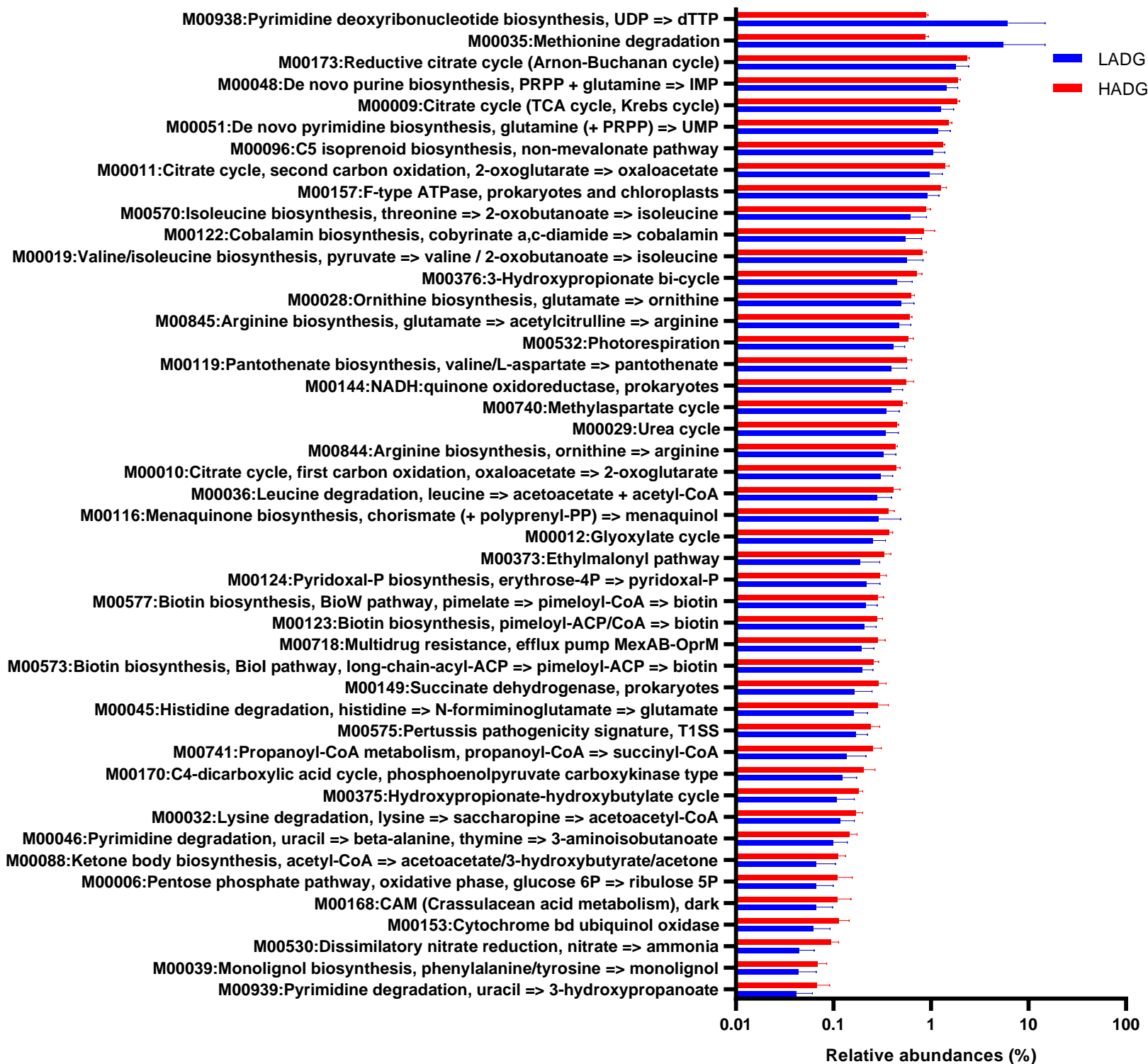

Supplement: Supplementary file 7 — Additional file 6: Figure S5. All significantly different KEGG modules in the rumen (LDA > 2, P < 0.05). [file 40168_2024_1844_MOESM6_ESM.pdf]

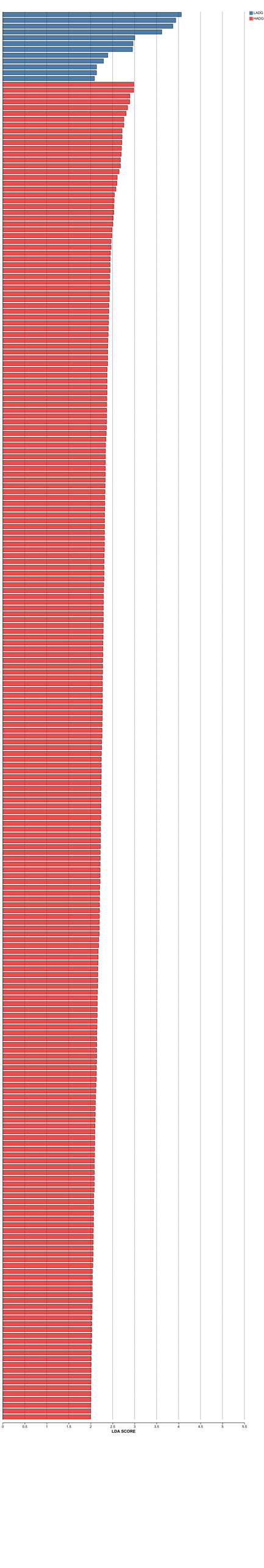

Supplement: Supplementary file 8 — Additional file 7: Figure S6. All significantly different KEGG enzymes in the rumen (LDA > 2, P < 0.05). [file 40168_2024_1844_MOESM7_ESM.pdf]

A

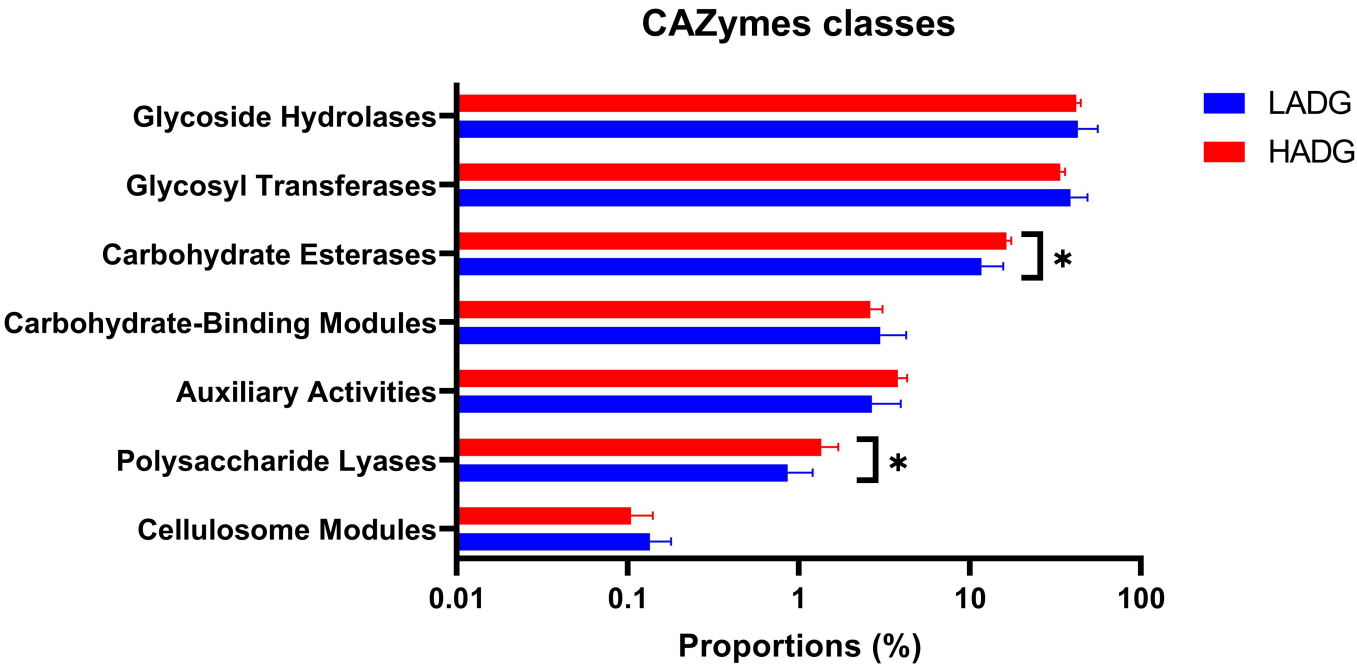

B

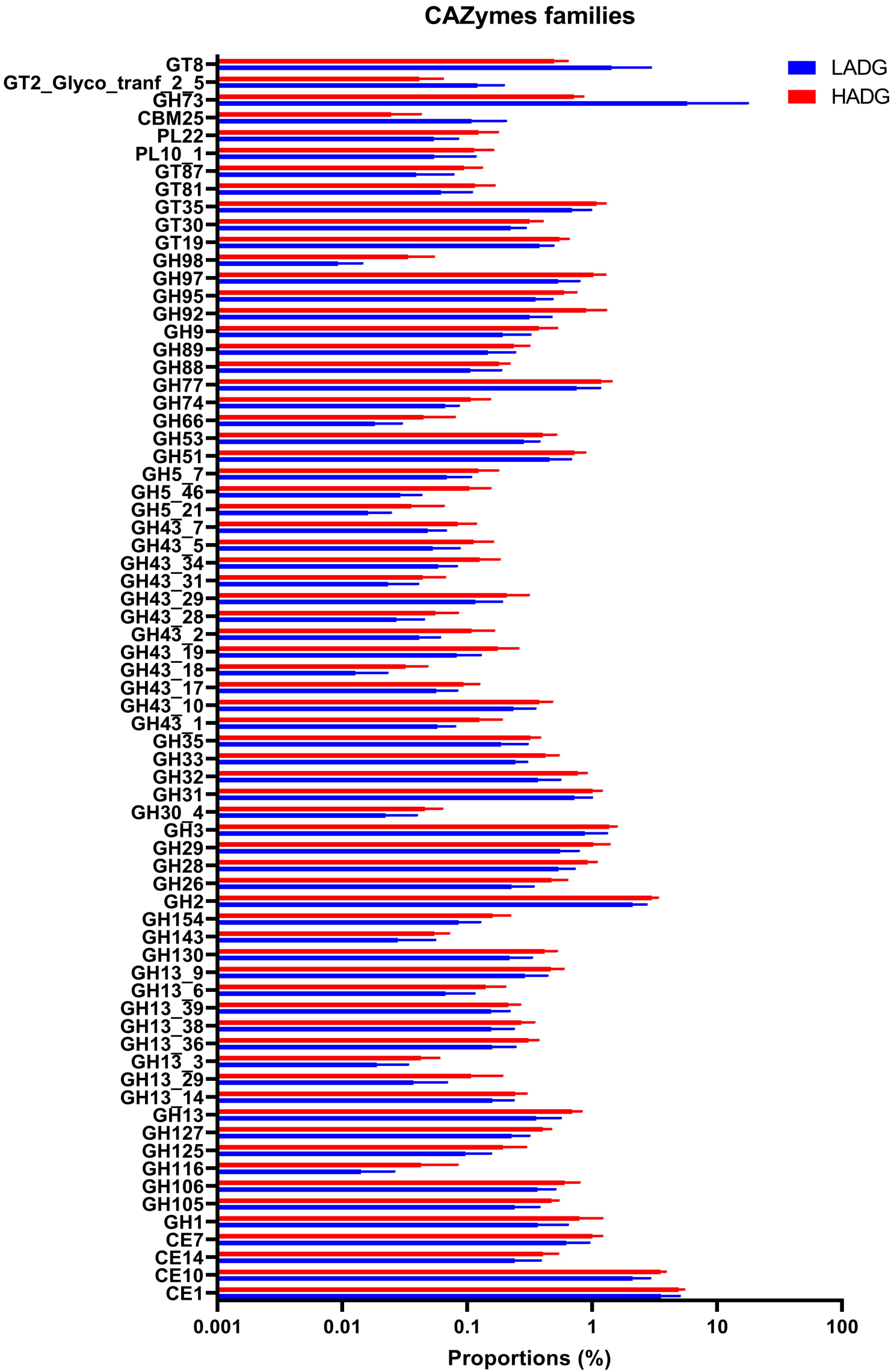

Supplement: Supplementary file 9 — Additional file 8: Figure S7. All significantly different CAZymes in the rumen. A) The class-level CAZymes profiles in the rumen. * means LDA > 2, P < 0.05. B) All significantly different family-level CAZymes in the rumen (LDA > 2, P < 0.05). [file 40168_2024_1844_MOESM8_ESM.pdf]

A

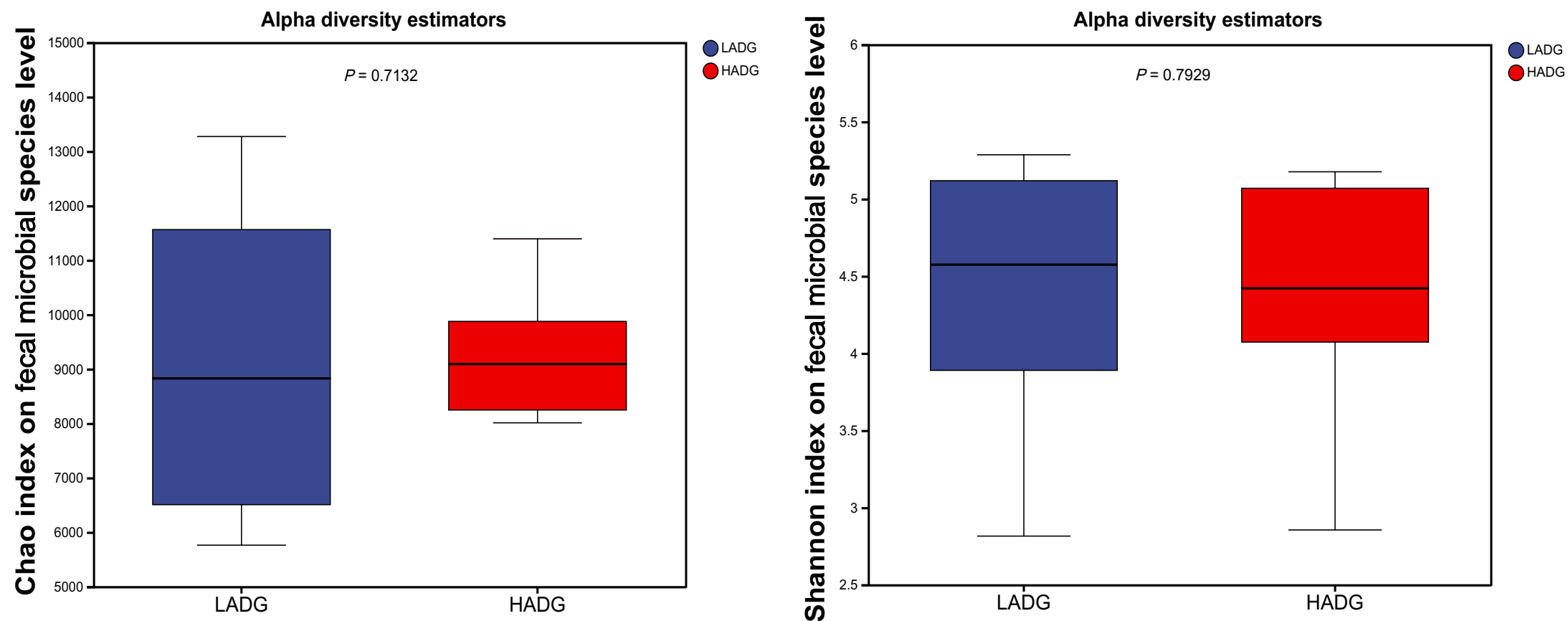

B

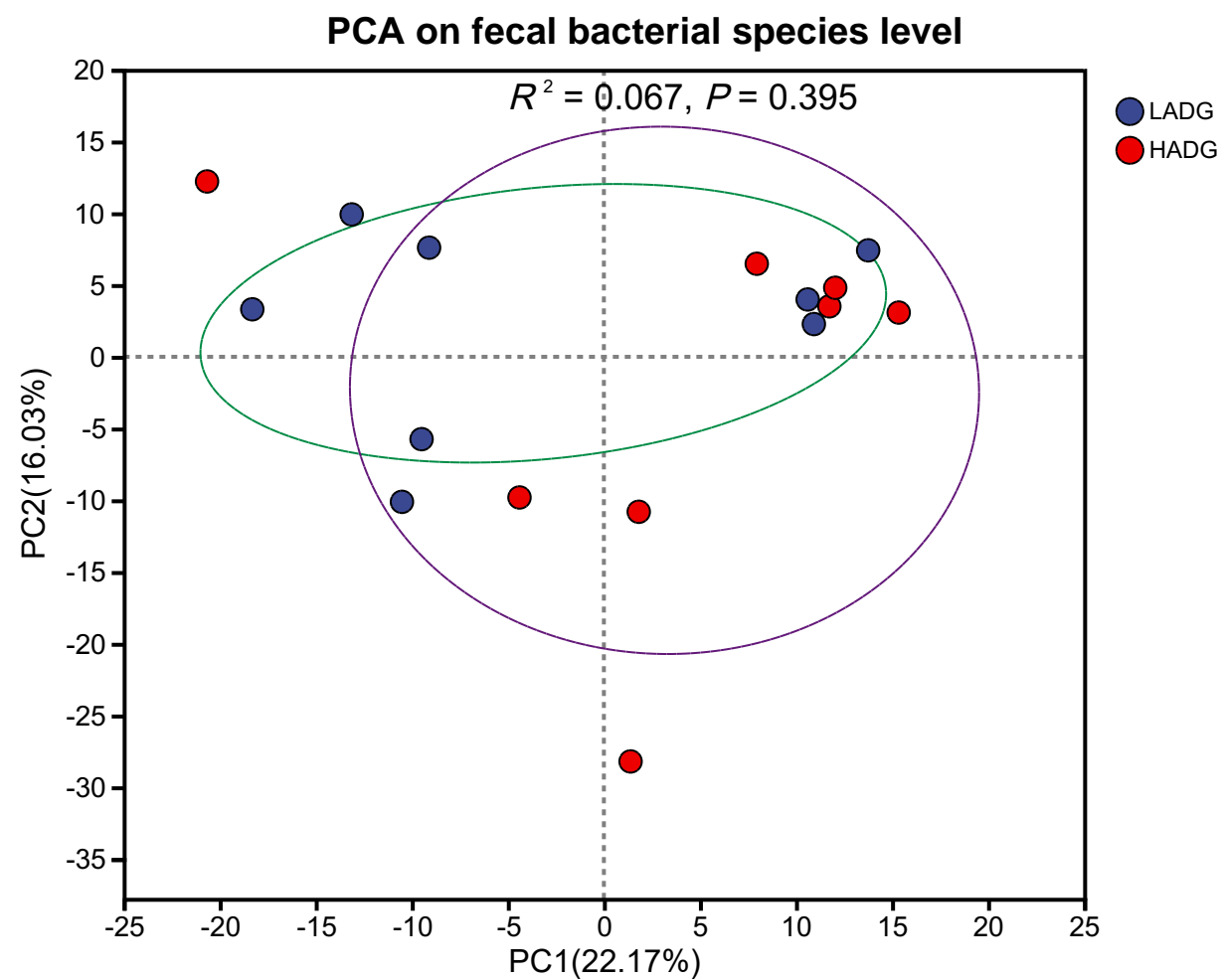

Supplement: Supplementary file 12 — Additional file 11: Figure S10. Alpha and beta diversity of fecal microbes between HADG and LADG calves. A) Alpha diversity (Chao and Shannon indices) of fecal microbes between HADG and LADG calves. B) The Principal Component Analysis (PCA) based on fecal bacterial species level between HADG and LADG calves. [file 40168_2024_1844_MOESM11_ESM.pdf]

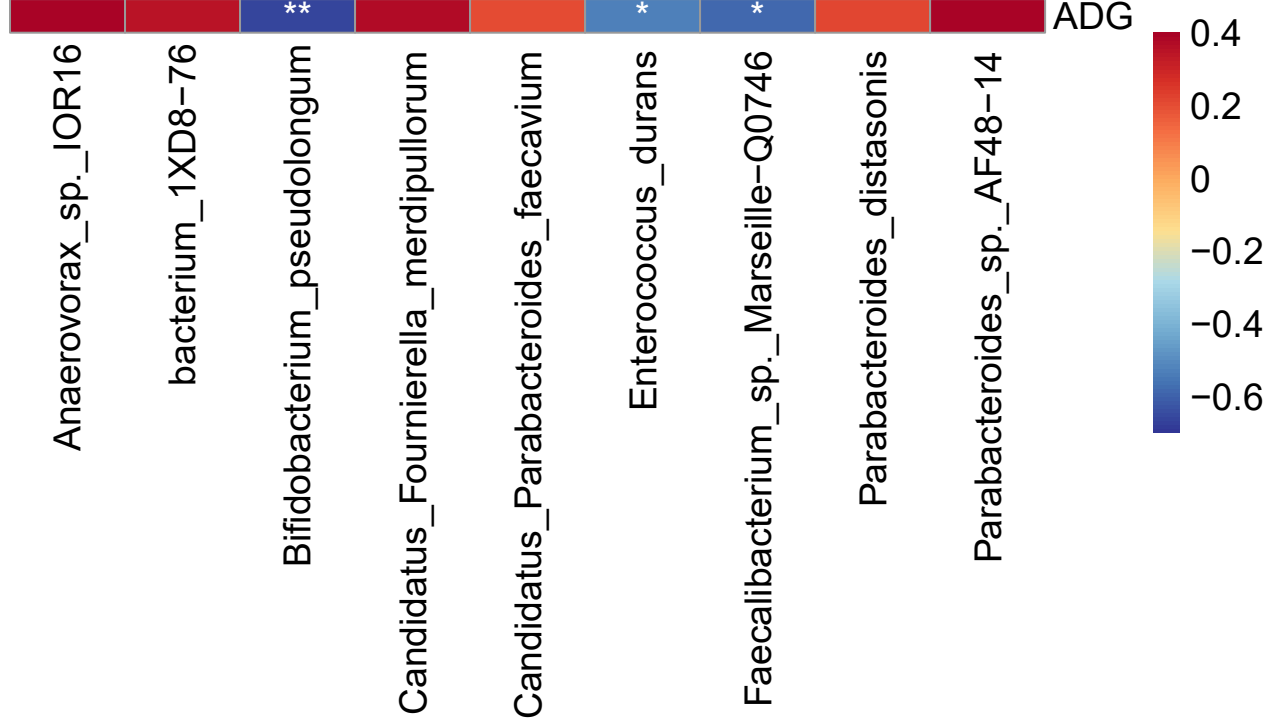

Supplement: Supplementary file 13 — Additional file 12: Figure S11. Heatmap displays the Spearman’s correlation coefficients among fecal microbes and host phenotypes. * means R > |0.50|, P < 0.05, ** means R > |0.50|, P < 0.01. [file 40168_2024_1844_MOESM12_ESM.pdf]

**A**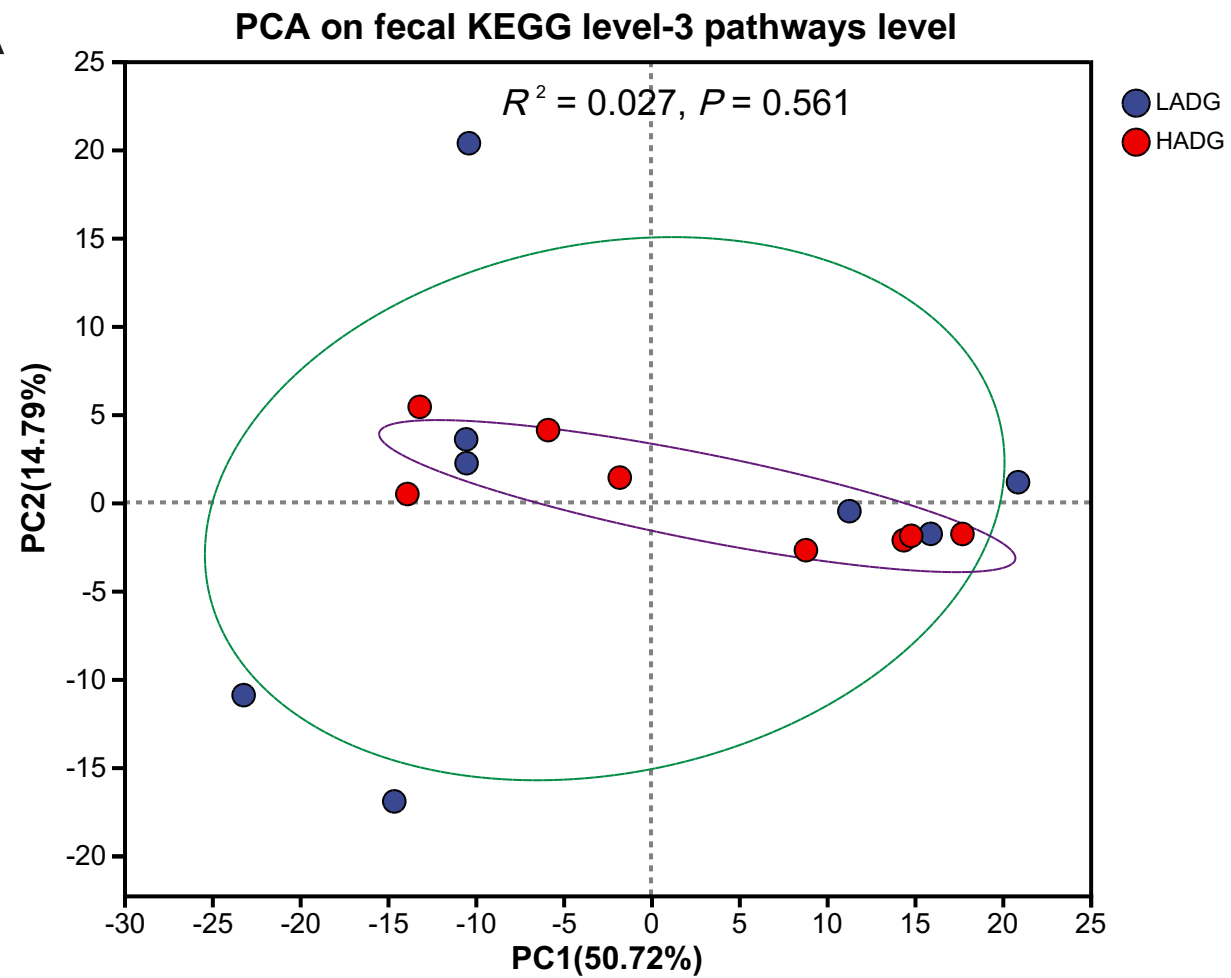**B**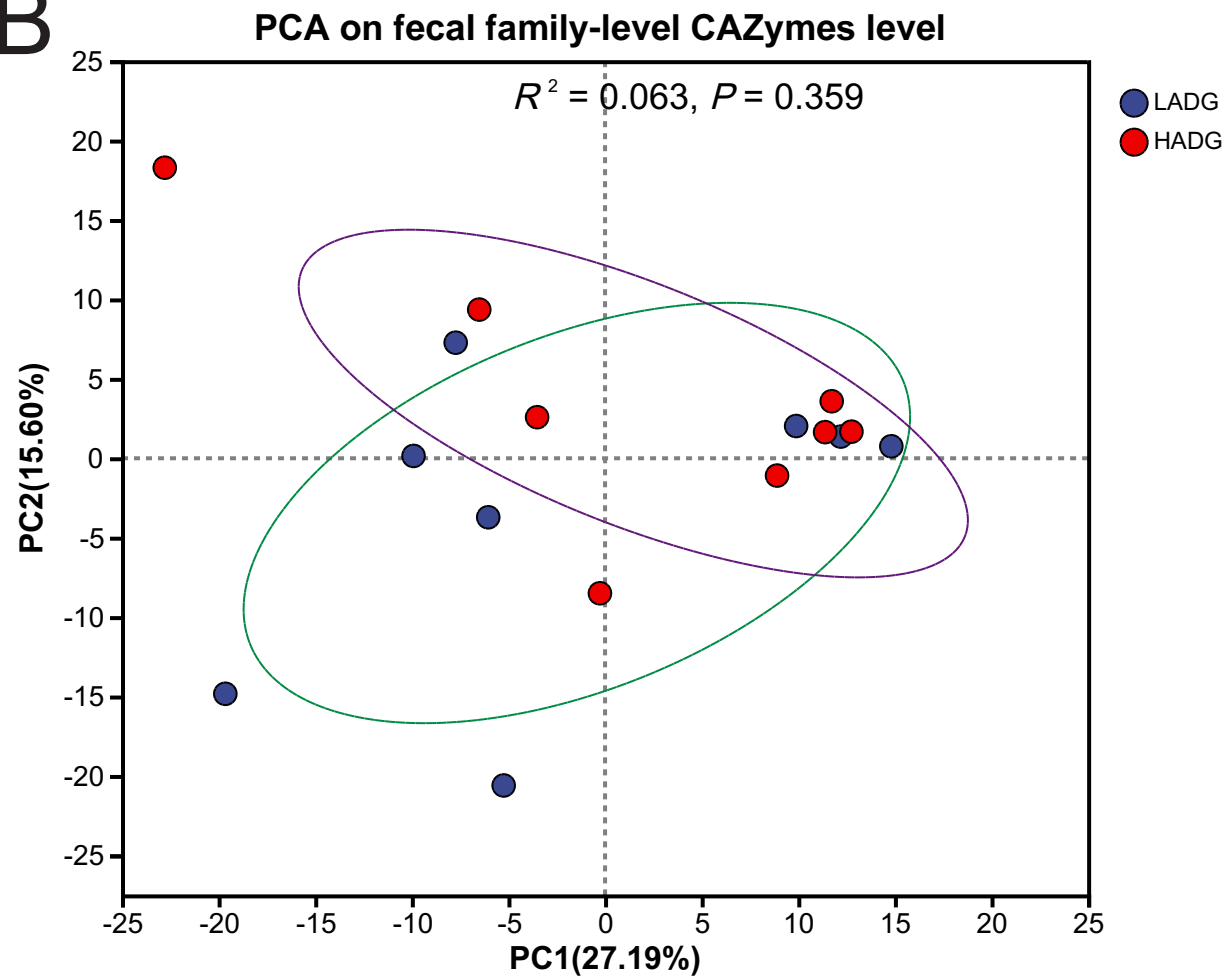

Supplement: Supplementary file 14 — Additional file 13: Figure S12. The Principal Component Analysis (PCA) profile of fecal microbial function between HADG and LADG calves. A) The Principal Component Analysis (PCA) profile of fecal KEGG level-3 pathways between HADG and LADG calves. B) The Principal Component Analysis (PCA) profile of fecal family-level CAZymes between HADG and LADG calves. [file 40168_2024_1844_MOESM13_ESM.pdf]

A

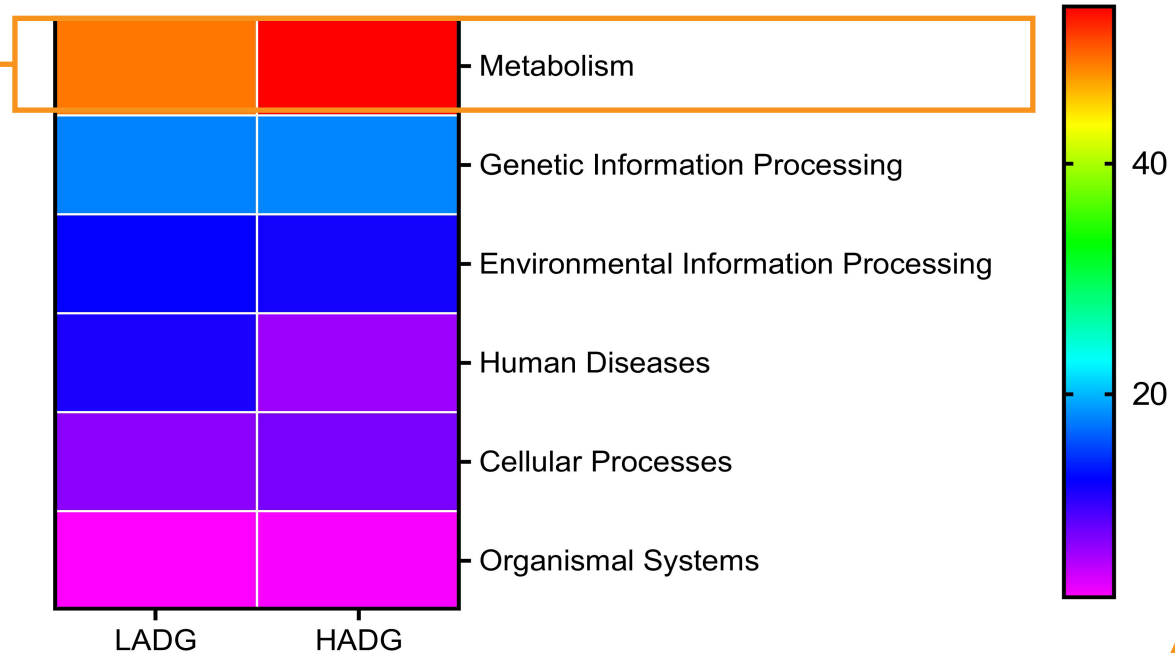

B

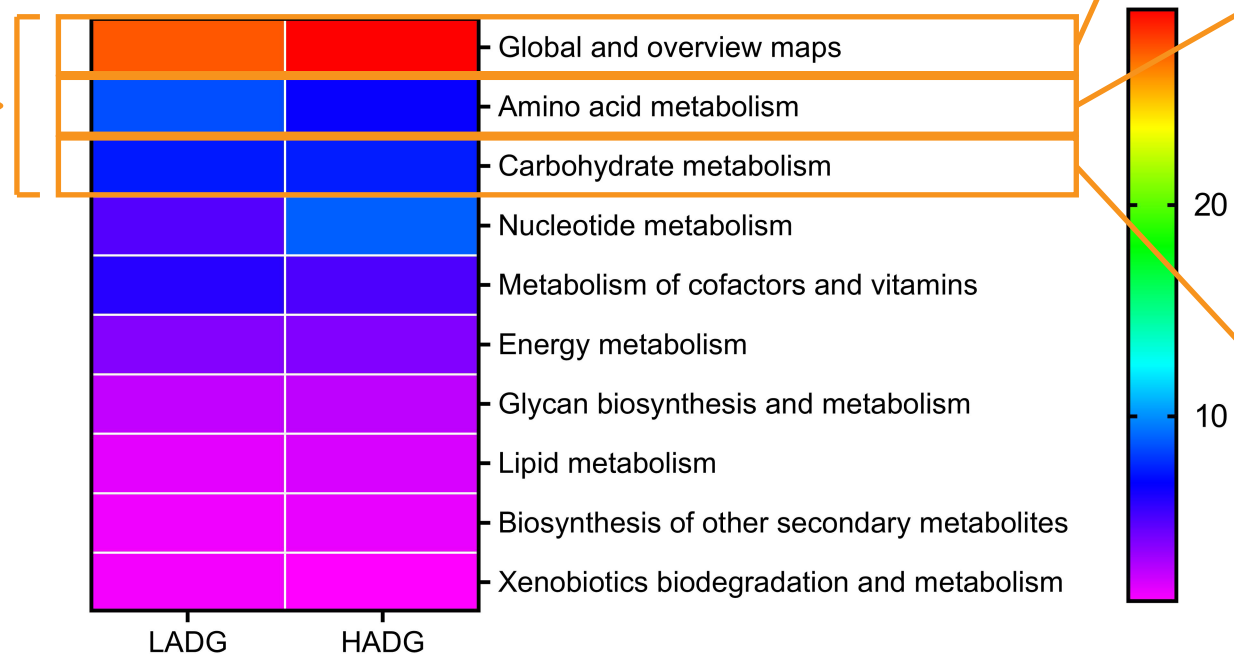

C

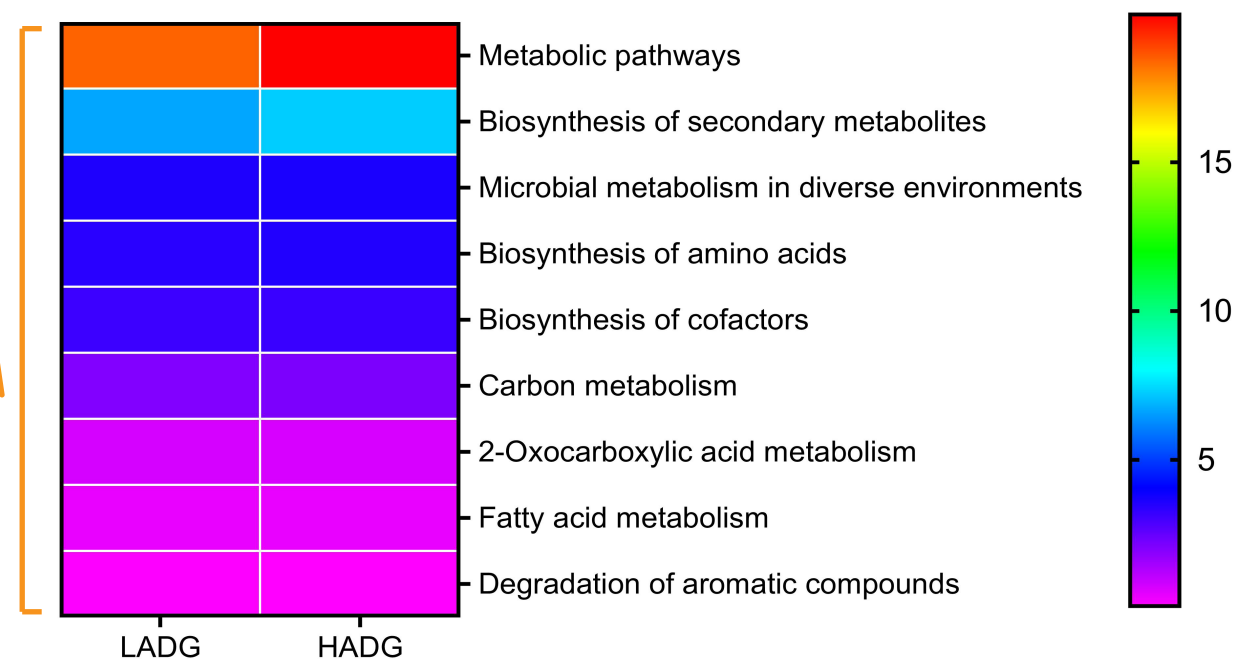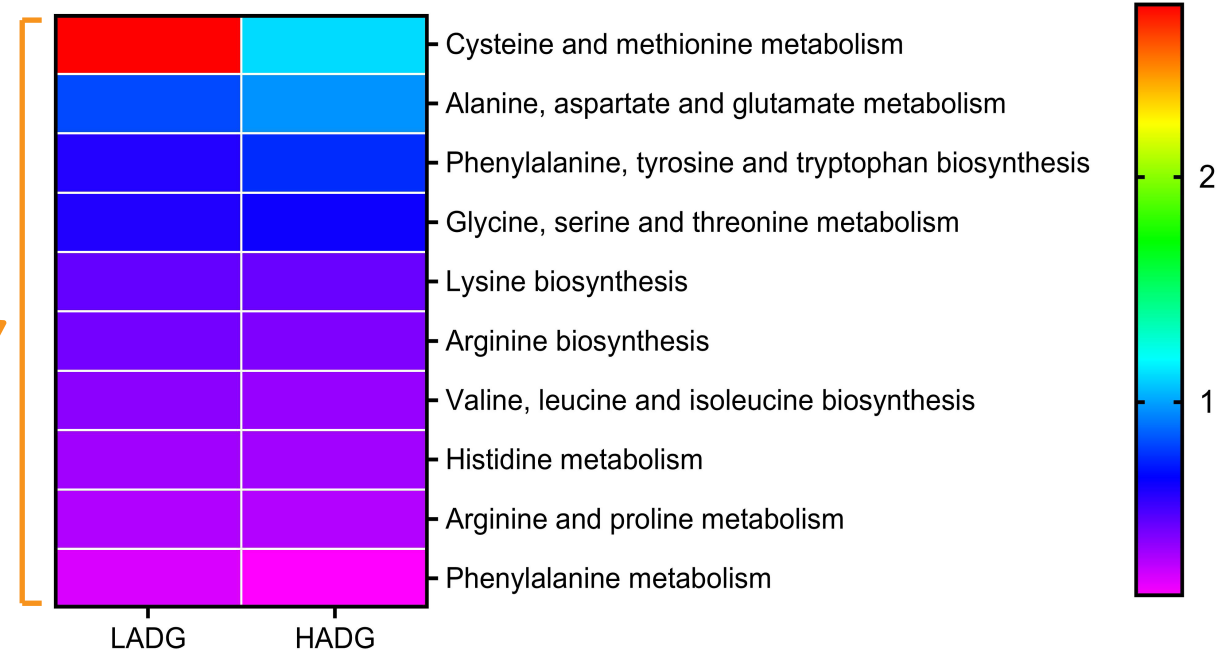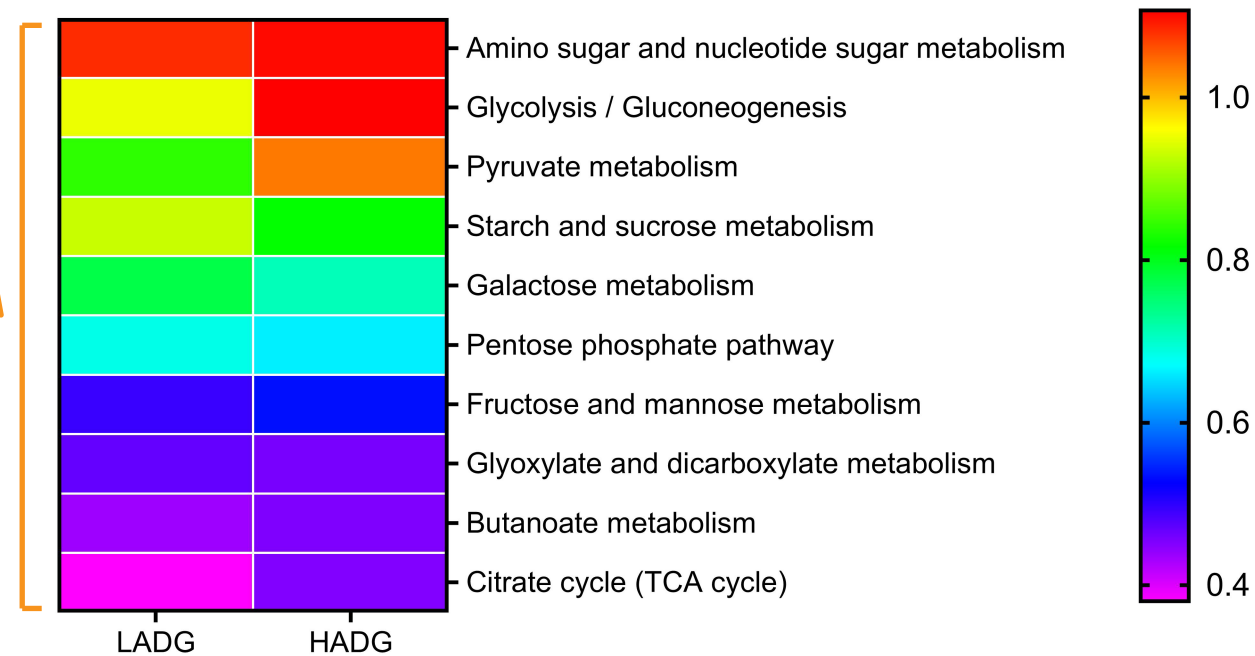

Supplement: Supplementary file 15 — Additional file 14: Figure S13. The distribution of KEGG pathways in the fecal microbiome. * means LDA > 2, P < 0.05. [file 40168_2024_1844_MOESM14_ESM.pdf]

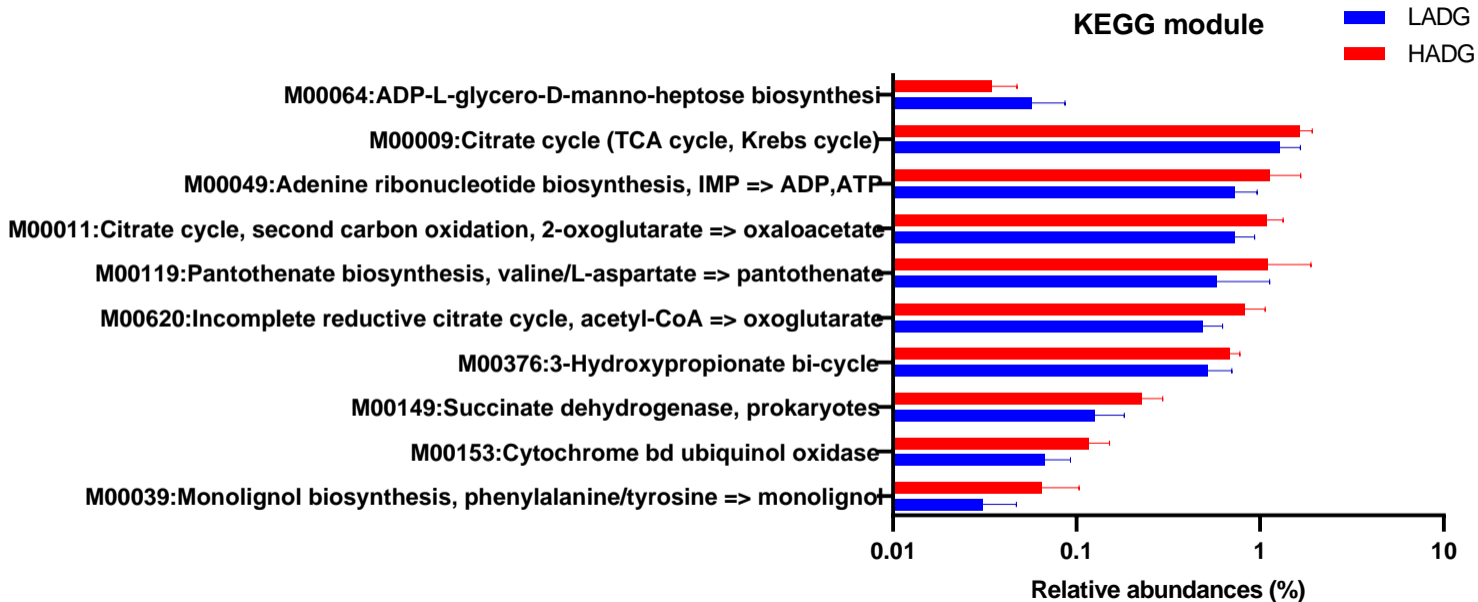

Supplement: Supplementary file 16 — Additional file 15: Figure S14. All significantly different KEGG modules in the feces (LDA > 2, P < 0.05). [file 40168_2024_1844_MOESM15_ESM.pdf]

A

## CAZymes classes

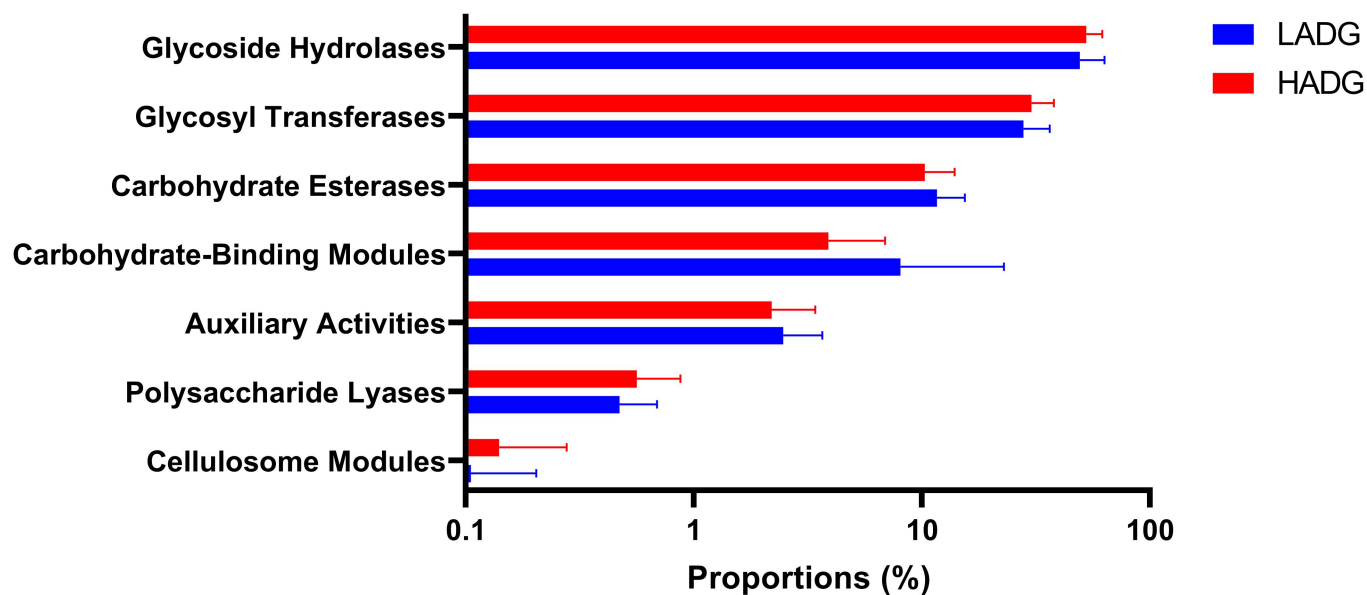

B

## CAZymes families

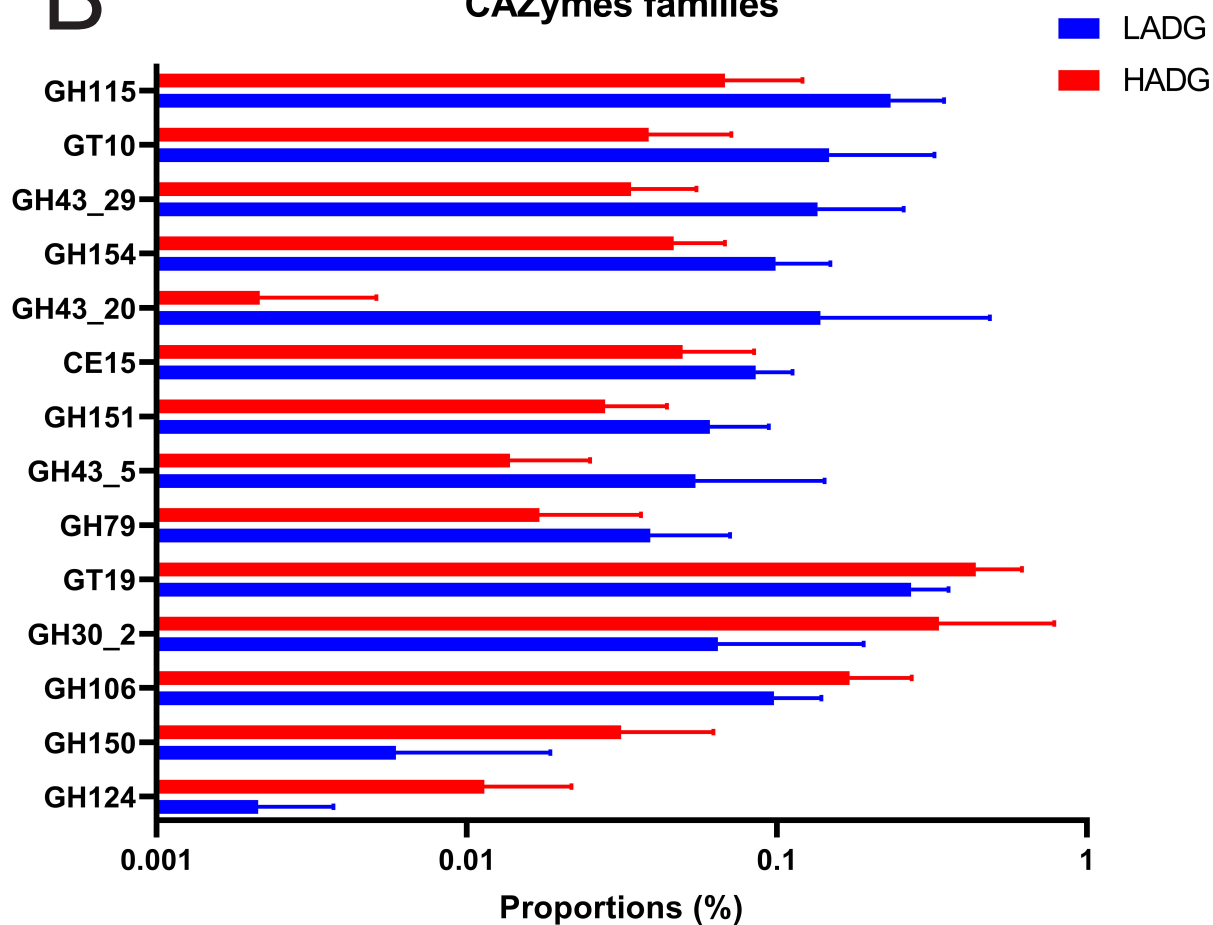

Supplement: Supplementary file 17 — Additional file 16: Figure S15. All significantly different CAZymes in the feces. A) The class-level CAZymes profiles in the feces. * means LDA > 2, P < 0.05. B) All significantly different family-level CAZymes in the feces (LDA > 2, P < 0.05). [file 40168_2024_1844_MOESM16_ESM.pdf]

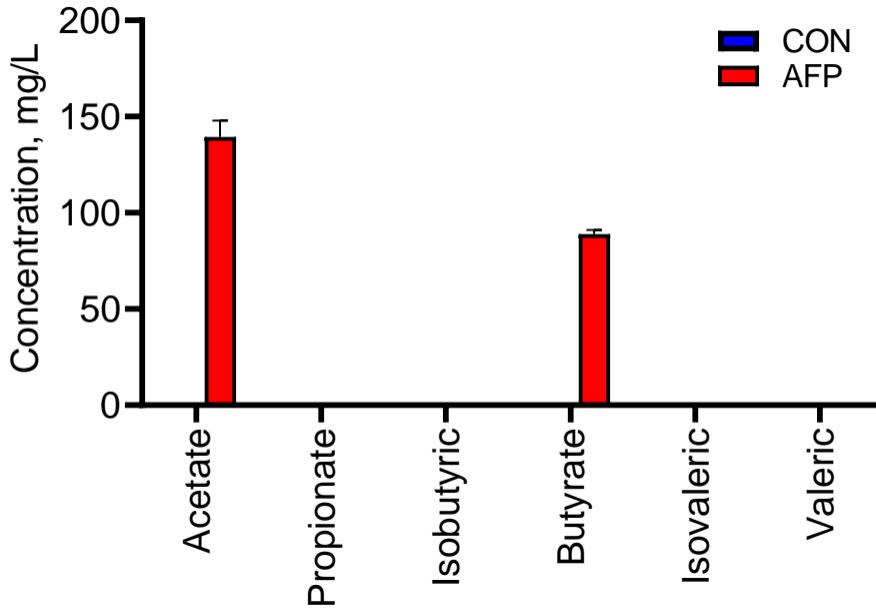

Supplement: Supplementary file 20 — Additional file 19: Figure S17. Acid production capacity of Acidaminococcus fermentans P41. CON means PYG liquid medium; AFP means PYG liquid medium + Acidaminococcus fermentans P41. [file 40168_2024_1844_MOESM19_ESM.pdf]

# A Substrate: Milk replacer

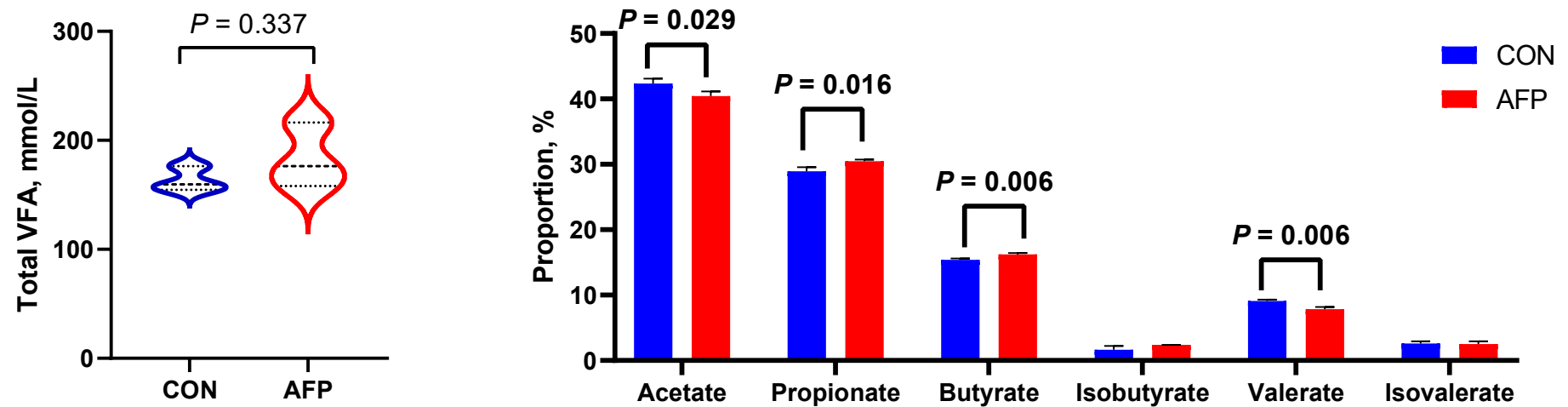

# B Substrate: Milk replacer + Starter

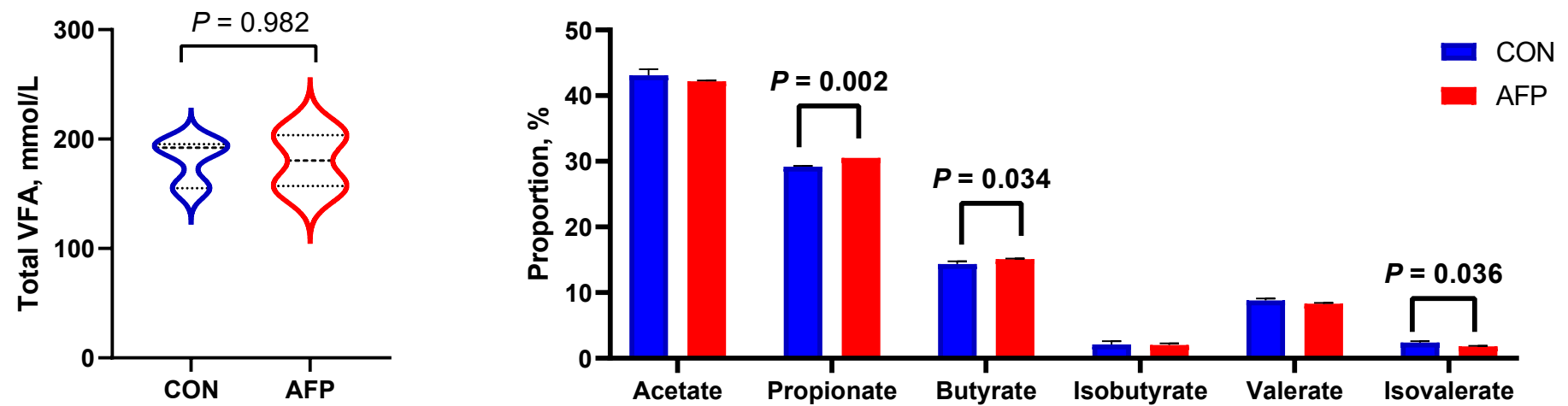

# C Substrate: Starter

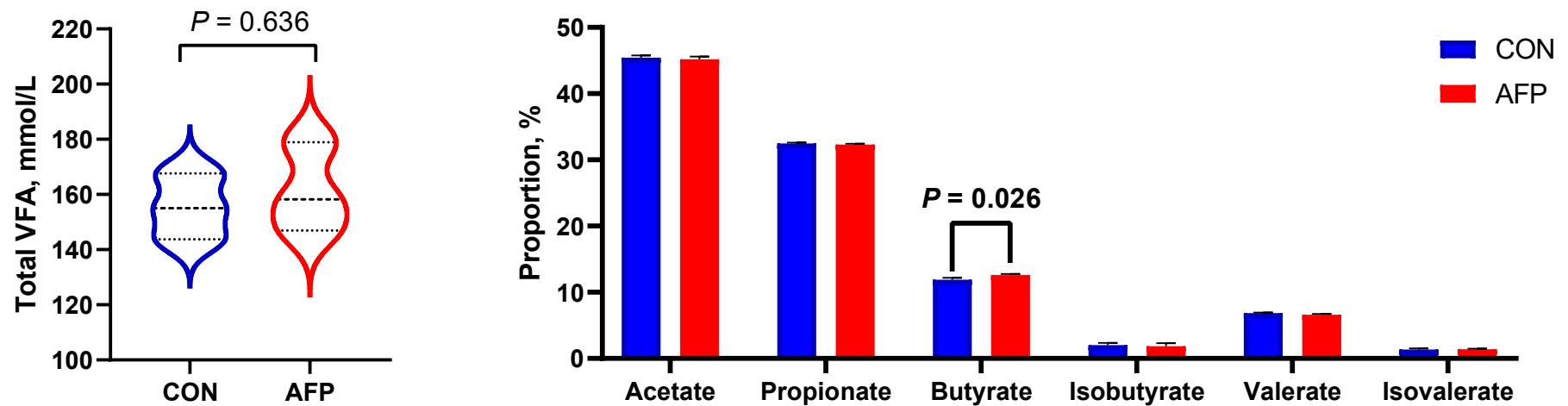

Supplement: Supplementary file 21 — Additional file 20: Figure S18. In vitro fermentation parameters. A) The in vitro fermentation parameters between the CON group and AFP group when using milk replacer as the substrate. B) The in vitro fermentation parameters between the CON group and AFP group when using milk replacer and starter as the substrate. C) The in vitro fermentation parameters between the CON group and AFP group when using starter as the substrate. CON means PYG liquid medium; AFP means PYG liquid medium + Acidaminococcus fermentans P41. [file 40168_2024_1844_MOESM20_ESM.pdf]
