# Supplementary material for: Rumen and hindgut microbiome regulate average daily gain of preweaning Holstein heifer calves in different ways
Source: Microbiome. 2024 Jul 19;12:131. doi: 10.1186/s40168-024-01844-7 (PMC11264748; doi:10.1186/s40168-024-01844-7)
Supplement: Supplementary file 10 — Additional file 9: Figure S8. Multiplex networks revealed how rumen microbes influence host phenotypes by regulating carbohydrate metabolism. Lines between two nodes represent the correlation, with a red line indicating a positive correlation and a blue line indicating a negative correlation (Spearman’s |r| > 0.50 and P < 0.05). [file 40168_2024_1844_MOESM9_ESM.pdf]

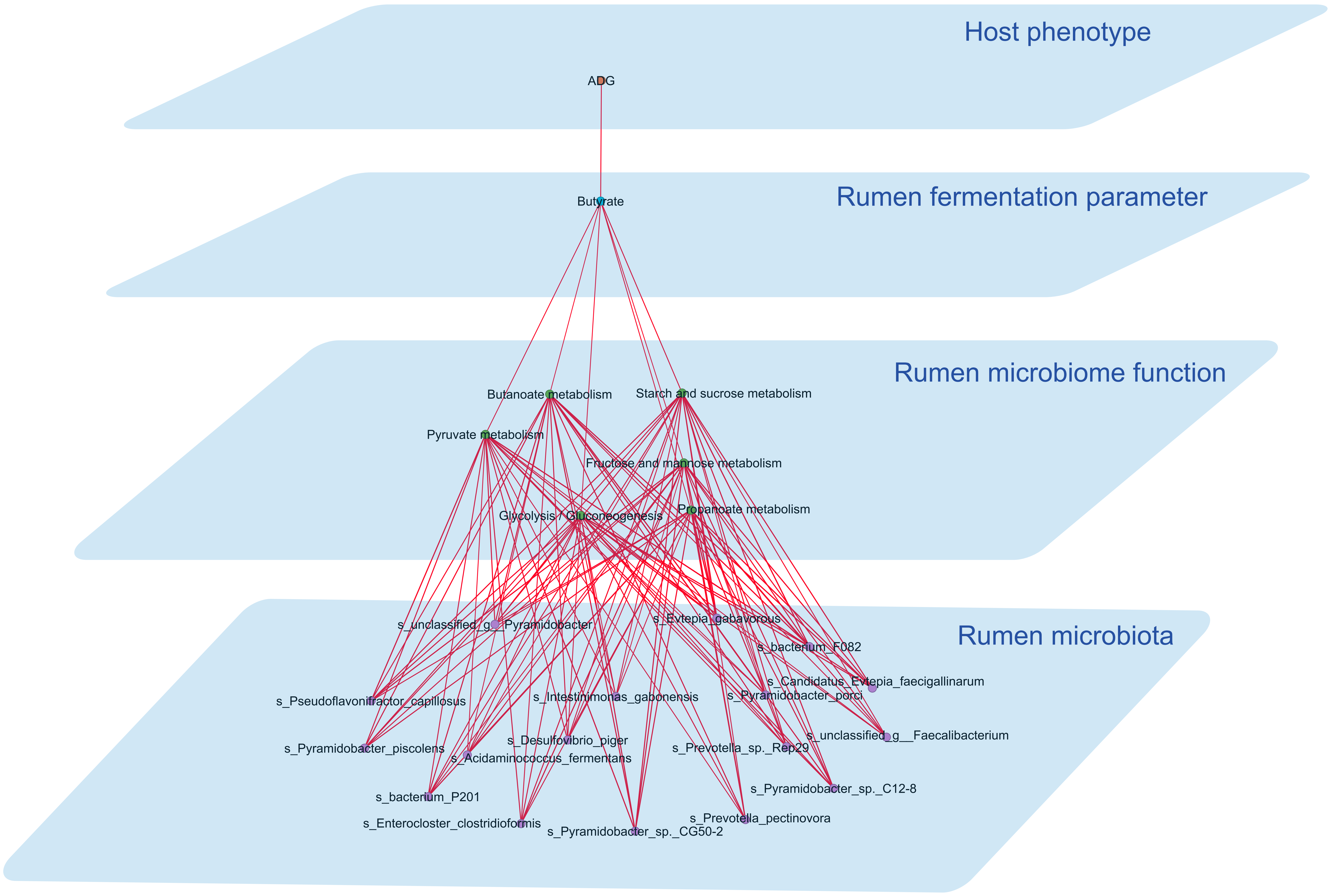
Host phenotype
ADG
Rumen fermentation parameter
Butyrate
Rumen microbiome function
Butanoate metabolism
Starch and sucrose metabolism
Pyruvate metabolism
Fructose and mannose metabolism
Glycolysis / Gluconeogenesis
Propanoate metabolism
Rumen microbiota
s_unclassified_g__Pyramidobacter
s_Evtepia_gabavorous
s_bacterium_F082
s_Candidatus_Evtepia_faecigallinarum
s_Pseudoflavonifractor_capillosus
s_Intestinimonas_gabonensis
s_Pyramidobacter_porci
s_unclassified_g__Faecalibacterium
s_Pyramidobacter_piscolens
s_Desulfovibrio_piger
s_Prevotella_sp._Rep29
s_Acidaminococcus_fermentans
s_Pyramidobacter_sp._C12-8
s_bacterium_P201
s_Prevotella_pectinovora
s_Enterocloster_clostridioformis
s_Pyramidobacter_sp._CG50-2
