# Supplementary material for: Rumen and hindgut microbiome regulate average daily gain of preweaning Holstein heifer calves in different ways
Source: Microbiome. 2024 Jul 19;12:131. doi: 10.1186/s40168-024-01844-7 (PMC11264748; doi:10.1186/s40168-024-01844-7)
Supplement: Supplementary file 11 — Additional file 10: Figure S9. Multiplex networks revealed how rumen microbes influence host phenotypes by regulating amino acid metabolism. Lines between two nodes represent the correlation, with a red line indicating a positive correlation and a blue line indicating a negative correlation (Spearman’s |r| > 0.50 and P < 0.05). [file 40168_2024_1844_MOESM10_ESM.pdf]

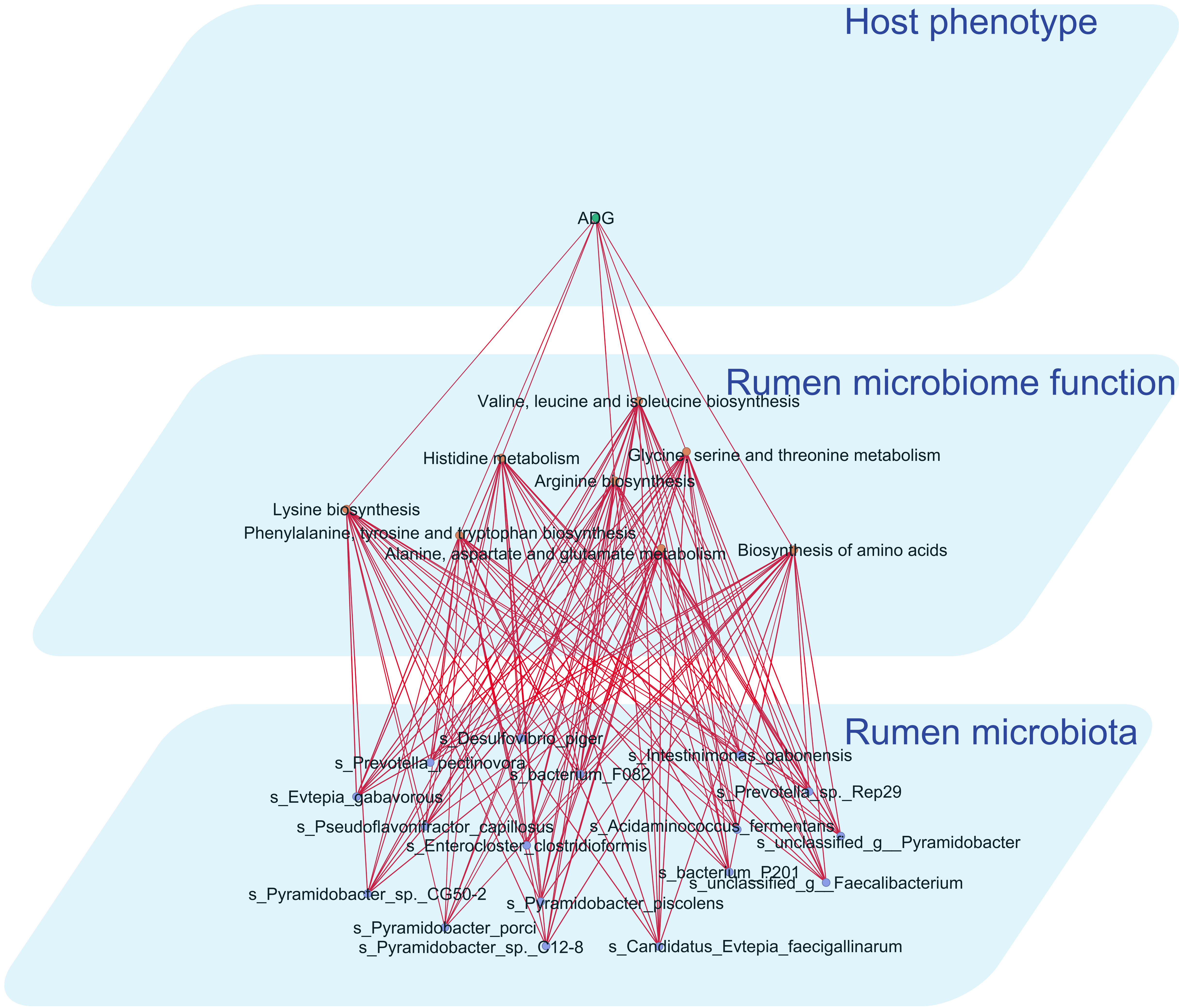
Host phenotype
ADG
Rumen microbiome function
Valine, leucine and isoleucine biosynthesis
Histidine metabolism
Glycine, serine and threonine metabolism
Arginine biosynthesis
Lysine biosynthesis
Phenylalanine, tyrosine and tryptophan biosynthesis
Alanine, aspartate and glutamate metabolism
Biosynthesis of amino acids
Rumen microbiota
s_Desulfovibrio_piger
s_Intestinimonas_gabonensis
s_Prevotella_pectinovora
s_bacterium_F082
s_Evtepia_gabavorous
s_Prevotella_sp._Rep29
s_Pseudoflavonifractor_capillosus
s_Acidaminococcus_fermentans
s_Enterocloster_clostridioformis
s_unclassified_g__Pyramidobacter
s_bacterium_P201
s_unclassified_g__Faecalibacterium
s_Pyramidobacter_sp._CG50-2
s_Pyramidobacter_piscolens
s_Pyramidobacter_porci
s_Pyramidobacter_sp._C12-8
s_Candidatus_Evtepia_faecigallinarum
