# Supplementary material for: Rumen and hindgut microbiome regulate average daily gain of preweaning Holstein heifer calves in different ways
Source: Microbiome. 2024 Jul 19;12:131. doi: 10.1186/s40168-024-01844-7 (PMC11264748; doi:10.1186/s40168-024-01844-7)
Supplement: Supplementary file 18 — Additional file 17: Table S19. 16s rRNA sequence of Acidaminococcus fermentans P41. [file 40168_2024_1844_MOESM17_ESM.docx]

Sequences:

TGCAGTCGAACGGAGAACTTTCTTCGGAATGTTCTTAGTGGCGAACGGGTGAGTAACGCGTAGGCAACCTGCCCTCTGGTTGGGGACAACATTCCGAAAGGGATGCTAATACCGAATGTGATCCTNNTTNCGCATGNAGNNAGGATGAAAGATGGCCTCTACTTGTAAGCTATCGCCAGAAGATGGGCCTGCGTCTGATTAGCTNGTAGGTGGGGTAACGGCTCACCTAGGCGATGATCAGTAGCCGGTCTGAGAGGATGAACGGCCACATTGGGACTGAGACACGGCCCAAACTCCTACGGGAGGCAGCAGTGGGGAATCTTCCGCAATGGACGAAAGTCTGACGGAGCAACGCCGCGTGAGTGATGAAGGTCTTCGGATTGTAAAACTCTGTTGTCAGGGACGAATGCACCGATNTATAATACANTTTGGTGTTGACGGTACCTGACGAGGAAGCCACGGCTAACTACGTGCCAGCAGCCGCGGTAATACGTAGGTGGCAAGCGTTGTCCGGAATTATTGGGCGTAAAGAGCATGTAGGCGGGCTTTTAAGTCCGACGTGAAAATGCGGGGCTTAACCCCGTATGGCGTTGGATACTGGGAGTCTTGAGTGCAGGAGAGGAAAGGGGAATTCCCAGTGTAGCGGTGAAATGCGTAGATATTGGGAGGAACACCAGTGGCGAAGGCGCCTTTCTGGACTGTGTCTGACGCTGAGATGCGAAAGCCAGGGTAGCAAACGGGATTAGATACCCCGGTAGTCCTGGCCGTAAACGATGGGTACTAGGTGTAGGAGGTATCGACCCCTTCTGTGCCGGAGTTAACGCAATAAGTACCCCGCCTGGGGACTACGATCGCAAGATTGAAACTCAAAGGAATTGACGGGGGCCCGCACAAGCGGTGGAGTATGTGGTTTAATTCGACGCAACGCGAAGAACCTTACCAAGGCTTGACATTGAGTGAAAGACCTAGAGATAGGTCCCTNTCTTCGGANNCACGAAAACAGGTGGTGCATGGCTGTCGTCAGCTCGTGTCGTGAGATGTTGGGTTAAGTCCCGCAACGAGCGCAACCCTTATCCTATGTTACCAGCACGCAACGGTGGGGACTCATAGGAGACTGCCAGGGATAACCTGGAGGAAGGCGGGGATGACGTCAAGTCATCATGCCCCTTATGTCTTGGGCTACACACGTACTACAATGGTCGGCAACAAAGGGCAGCGAAGCCGCGAGGCGGAGCCAATCCCAGAAACCCGACCCCAGTTCGGATCGCAGGCTGCAACCCGCCTGCGTGAAGTTGGAATCGCTAGTAATCGCAGGTCAGCATACTGCGGTGAATACGTTCCCGGGCCTTGTACACACCGCCCGTCACACCACGAAAGTTGGTAACACCCGAAGCCGGTGAGATAACCTTTTAG
