# Supplementary material for: Rumen and hindgut microbiome regulate average daily gain of preweaning Holstein heifer calves in different ways
Source: Microbiome. 2024 Jul 19;12:131. doi: 10.1186/s40168-024-01844-7 (PMC11264748; doi:10.1186/s40168-024-01844-7)
Supplement: Supplementary file 19 — Additional file 18: Figure S16. Genomic architecture of Acidaminococcus fermentans P41. Each circle, from centre to the outside, represents the following features. The first circle represents the scale mark, the second circle represents GC skew, the third circle represents GC content, the fourth and seventh circles represent every COG to which each coding sequence (CDS) belongs, and the fifth and sixth circles represent the locations of CDS, tRNA and rRNA in the genome. [file 40168_2024_1844_MOESM18_ESM.pdf]

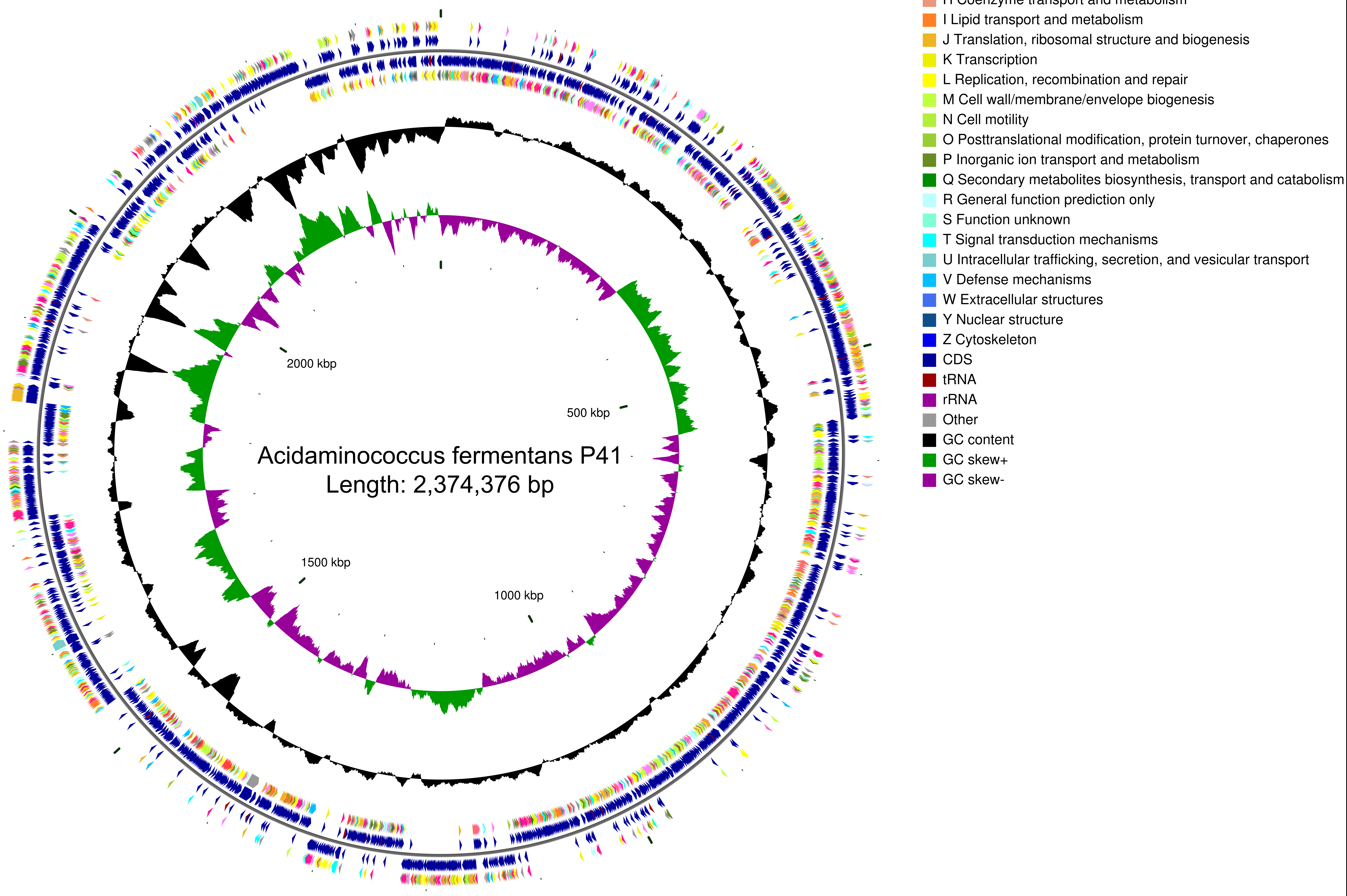

Acidaminococcus fermentans P41
Length: 2,374,376 bp
500 kbp
1000 kbp
1500 kbp
2000 kbp
A RNA processing and modification
B Chromatin structure and dynamics
C Energy production and conversion
D Cell cycle control, cell division, chromosome partitioning
E Amino acid transport and metabolism
F Nucleotide transport and metabolism
G Carbohydrate transport and metabolism
H Coenzyme transport and metabolism
I Lipid transport and metabolism
J Translation, ribosomal structure and biogenesis
K Transcription
L Replication, recombination and repair
M Cell wall/membrane/envelope biogenesis
N Cell motility
O Posttranslational modification, protein turnover, chaperones
P Inorganic ion transport and metabolism
Q Secondary metabolites biosynthesis, transport and catabolism
R General function prediction only
S Function unknown
T Signal transduction mechanisms
U Intracellular trafficking, secretion, and vesicular transport
V Defense mechanisms
W Extracellular structures
Y Nuclear structure
Z Cytoskeleton
CDS
tRNA
rRNA
Other
GC content
GC skew+
GC skew-
